# Loddigesiinols G–J: α-Glucosidase Inhibitors from *Dendrobium loddigesii*

**DOI:** 10.3390/molecules19068544

**Published:** 2014-06-23

**Authors:** Yu Lu, Ming Kuang, Gu-Ping Hu, Rui-Bo Wu, Jun Wang, Lan Liu, Yong-Cheng Lin

**Affiliations:** 1 School of Pharmaceutical Sciences, Sun Yat-sen University, Guangzhou 510006, China; E-Mails: luyu0626@sina.com (Y.L.); wurb3@mail.sysu.edu.cn (R.-B.W.); 2 Guangdong Pharmaceutical University, Guangzhou 510006, China; E-Mail: kuangm55555@gmail.com; 3 School of Chemistry and Chemical Engineering, Sun Yat-sen University, Guangzhou 510275, China; E-Mails: huguping@mail.sysu.edu.cn (G.P.H.); cesllan@mail.sysu.edu.cn (L.L.); ceslyc@mail.sysu.edu.cn (Y.-C.L.)

**Keywords:** *Dendrobium loddigesii*, *α*-glucosidase inhibitory activity, polyphenols

## Abstract

Four new polyphenols, loddigesiinols G–J (compounds **1**–**4**) and a known compound, crepidatuol B (**5**), were isolated from the stems of *Dendrobium loddigesii* that have long been used in Traditional Chinese Medicine and have recently been used to treat type 2 diabetes. Compounds **1**–**5** structures were elucidated based on spectroscopic analysis. The absolute configurations of compounds **1**–**4** were determined using theoretical calculations of electronic circular dichroism (ECD), and the absolute configuration of compound **5** was determined by a comparison of the experimental ECD spectra and the literature data. Compounds **1**–**5** are strong inhibitors of *α*-glucosidase, with IC_50_ values of 16.7, 10.9, 2.7, 3.2, and 18.9 μM, respectively. Their activities were significantly stronger than *trans*-resveratrol as a positive control (IC_50_ values of 27.9 μM).

## 1. Introduction

Approximately 80 *Dendrobium* species (Orchidaceae), called “Shi Hu” in Chinese, are distributed across China; 50 of these species have long been used in Traditional Chinese Medicine [1,2]. *D. loddigesii* is a perennial herb that is abundant in southern and southwest China [1,2]. The stems of *D. loddigesii*, which are the most important “Shi Hu” crude drug, have been used for the treatment of gastrosis, fever, and dizziness [3]. This herb has also recently been used to treat type 2 diabetes. Animal and human studies have shown anti-diabetic effects of *D. loddigesii* stem extract (the traditional Chinese medicine apozem) [4,5,6]. To date, chemical studies of *D. loddigesii* have yielded bibenzyls, phenanthrenes, alkaloids, and lignans [7,8,9], but the chemical constituents of *D. loddigesii* that are responsible for lowering blood glucose levels have not been reported. Recently we have initiated a program of phytochemical and biological studies of the stems of *D. loddigesii*; many known compounds were isolated, seven of which had been reported [10]. Here, we report again the isolation and structural elucidation of four new polyphenols (compounds **1**–**4**), and a known compound (compound 5) from *D. Loddigesii* and show that these compounds inhibit *α*-glucosidase activity *in vitro*. It is noteworthy that compounds **3** and **4** were 10 times more potent than *trans*-resveratrol (IC_50_ value of 27.9 μM) [11,12].

## 2. Results and Discussion

**Figure 1:**
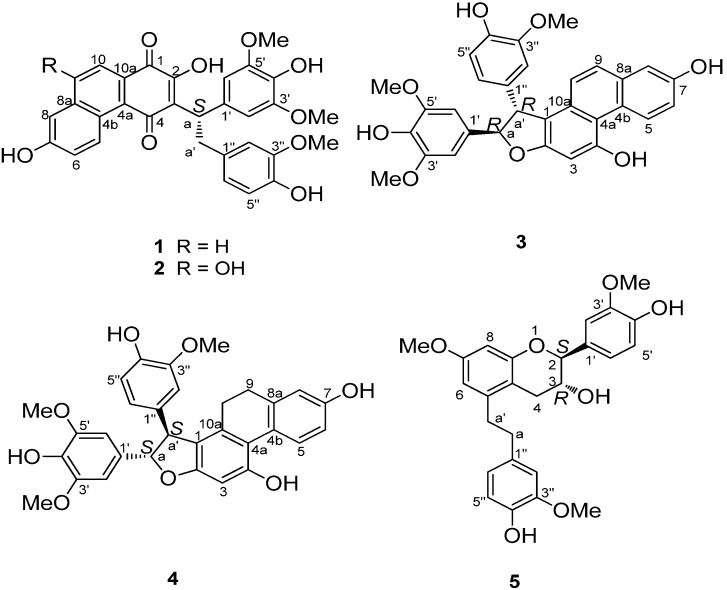
The chemical structures of compounds **1**–**5**.

**Figure 2:**
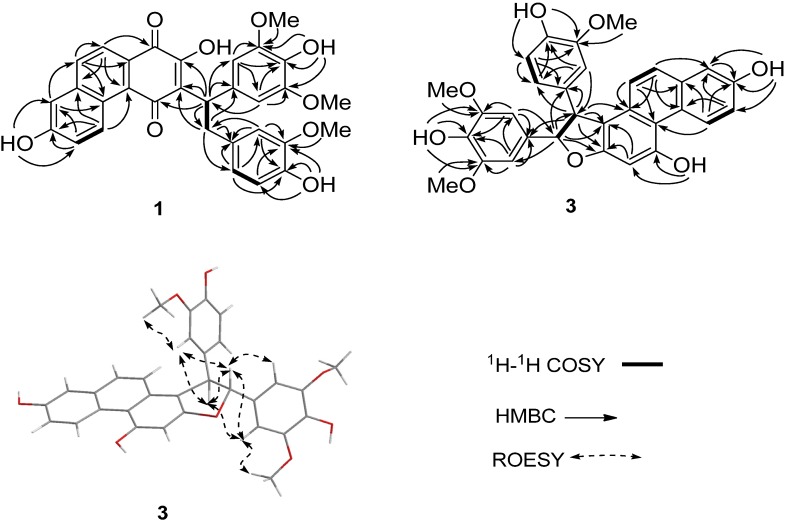
^1^H-^1^H COSY and HMBC correlations of compounds **1** and **3**.

**Figure 3:**
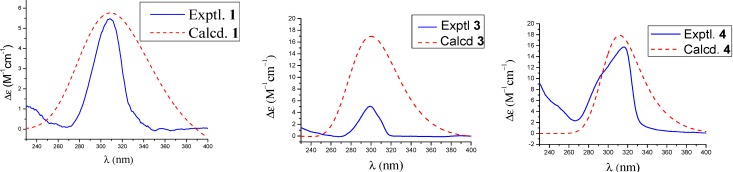
Calculated and experimental ECD spectra of **1**, **3** and **4**.

Loddigesiinol G (compound **1**, Figure 1) was obtained as a red amorphous solid and had a molecular formula of C_31_H_26_O_9_ as determined by HRESIMS data (observed *m/z* 541.15024 [M-H]^−^, calculated 541.15041). The ^13^C-NMR and DEPT spectra (Table 1) indicated the presence of two carbonyl groups, two probable quinone carbonyls (183.0 and 189.5), 24 olefinic carbons, one sp^3^ CH_2_ group, one sp^3^ CH group, and three MeO groups. The ^1^H-NMR and ^1^H-^1^H COSY spectra (Table 1) showed the signals of three pairs of ABX spin systems (δ 6.85/6.60/6.71, 9.39/7.35/7.26, and 7.96/7.93/7.26), a two-proton singlet at δ 6.90 in the aromatic region, and one group signal of three coupled-protons (δ_H_ 4.81/3.36/3.66). In the HMBC spectrum (Figure 2), rich correlation data allowed us to unambiguously establish a 1,4-phenanthrenedione segment and a bibenzyl moiety. In addition, the HMBC multiple correlations from H-a to C-2, C-3, and C-4, and from H-a' to C-3 revealed the connection of the bibenzyl and 1,4-phenanthrenedione substructures between C-3 and C-a. Two of the three MeO signals overlapped at δ_H_ 3.80, and their protons correlated with C-3' and C-5', respectively; the other MeO at δ_H_ 3.67 correlated with C-3''. Therefore, these MeO groups are located in the C-5', C-3', and C-3'' positions of the bibenzyl segment. Based on the HMBC correlations, three hydroxyl groups at δ_H_ 9.36, 7.21, and 7.02 were easily assigned to C-7, C-4', and C-4'', respectively, but the remaining hydroxyl group was not observed in the ^1^H-NMR spectrum; based on the chemical shift, it could only be located at C-2. The experimental ECD spectra of compound **1** showed a positive Cotton effect at 309 nm. The calculated ECD of **1** in a *S*-configuration matched well with the experimental data (Figure 3). Thus, the absolute configuration of **1** was assigned as a *S-*configuration.

**Table 1:** NMR data for compounds **1**–**2**.

No.	1 *^a^*		2 *^b^*	
	*δ_H_* (*J*)	*δ_c_*	*δ_H_* (*J*)	*δ_c_*
1		183.0, C		183.4, C
2		153.4, C		152.9, C
3		126.8, C		126.4, C
4		189.5, C		188.6, C
4a		140.8, C		122.1, C
4b		125.4, C		127.7, C
5	9.39, d (9.6)	131.6, CH	9.45, d (9.5)	131.5, CH
6	7.35, d (9.6)	123.0, CH	7.33, dd (9.5, 3.0)	122.8, CH
7		158.7, C		157.9, C
8	7.26, brs	111.0, CH	7.63, brs	105.4, CH
8a		129.7, C		131.4, C
9	7.96, d (8.4)	132.9, CH		157.2, C
10	7.93, d (8.4)	122.3, CH	7.38, s	103.8, CH
10a		128.1, C		129.8, C
1'		134.6, C		134.8, C
2'	6.90, s	107.4, CH	6.89, s	107.4, CH
3'		148.5, C		148.4, C
4'		135.6, C		135.5, C
5'		overlap with 3'		overlap with 3'
6'	overlap with 2'	overlap with 2'	overlap with 2'	overlap with 2'
1''		133.2, C		133.3, C
2''	6.85, s	113.4, CH	6.85, d (2.0)	113.3, CH
3''		148.0, C		147.9, C
4''		145.7, C		145.6, C
5''	6.60, d (7.8)	115.6, CH	6.61, d (8.0)	115.5, CH
6''	6.71, d (7.8)	122.3, CH	6.70, dd (8.0, 2.0)	122.2, CH
A	4.81, dd (10.2, 6.6)	43.9, CH	4.80, dd (10.0, 6.5)	43.7, CH
a'*α*	3.36, dd (13.8, 6.6)	38.7, CH_2_	3.67, dd (13.5, 10.0)	38.7, CH_2_
a'*β*	3.66, dd (13.8, 10.2)		3.34, dd (13.5, 7.0)	
3'-CH_3_O	3.80, s	56.8, CH_3_	3.80, s	56.8, CH_3_
3''-CH_3_O	3.67, s	56.2, CH_3_	3.67, s	56.1, CH_3_
5'-CH_3_O	overlap with 3'	overlap with 3'	overlap with 3'	overlap with 3'
2-OH	unobserved		unobserved	
7-OH	9.36, brs		unobserved	
9-OH			unobserved	
4''-OH	7.21, brs		unobserved	
4'-OH	7.02, brs		unobserved	

The data were recorded at *^a^* 600 MHz (^1^H-NMR) and 150 MHz (^13^C-NMR); or *^b^* 500 MHz (^1^H-NMR) and 125 MHz (^13^C-NMR); chemical shifts (*δ*) are in ppm and coupling constants (*J*) are in Hz.

Loddigesiinol H (compound **2**, Figure 1) has a molecular formula of C_31_H_26_O_10_ based on HRESIMS data (observed *m/z* 557.14544 [M-H]^−^, calculated 557.14532), with one more oxygen atom than compound **1**. The ^1^H- and ^13^C-NMR spectra of compound **2** were very similar to those of compound 1 (Table 1), except for the absence of a doublet signal at 7.96 (d, *J* = 8.4 Hz) and the change of a doublet signal at 7.93 (d, *J* = 8.4 Hz) to singlet signal at 7.38 (s) in the aromatic region. These results suggested that compound **2** with an added OH group. The ^1^H-^1^H COSY and HMBC correlations of compound **2** were also similar to those of **1** (Figure S24 and Table S2, Supporting Information); these results confirmed that the location of the additional OH group was at C-9 based on the 2D NMR spectra. The absolute configuration of compound **2** is also *S* (like compound **1**), based on the same strong positive Cotton effect at 308 nm (Figure S11, Supporting Information) and the same chromophore in compounds **2** and **1**.

Loddigesiinol I (compound **3**, Figure 1) was assigned the molecular formula of C_31_H_26_O_8_ based on HRESIMS data (observed *m/z* 525.15528 [M-H]^−^, calculated 525.15549), requiring 19 degrees of unsaturation. The ^13^C-NMR and DEPT spectra of compound **3** (Table 2) revealed the presence of three MeO groups, one CH group, one oxygenated CH group, and 26 olefinic carbons, accounting for 13 of the 19 degrees of unsaturation required by the molecular formula. These data suggested that compound **3** was a six-ring compound. The ^1^H-NMR spectra (Table 2) displayed the signals of three groups of ABX spin systems (δ_H_ 6.87/6.79/6.67, 9.61/7.15/7.16 and 7.16/7.39/7.20), a two-aromatic-proton singlet at δ_H_ 6.89, and two sp^3^*ortho*-methine groups (*δ*_H_ 5.49/4.86). In the HMBC spectrum (Figure 2), the correlations of H-a to C-1', of H-a' to C-1'', of H-5 and H-10 to C-4a, of H-9 to C-8 and C-10a allowed the assignment of C and H of a phenanthrene segment and a bibenzyl moiety, which was similar to those of compounds **1** and **2**. The HMBC spectrum enabled the determination of the overall structure of compound **3**; in particular, the multiple correlations between H-a and C-1, C-2, and C-a', and between H-a' and C-1 and C-2 revealed the structural portion of a furan ring. The *trans* relationship between H-a and H-a' was assigned based on the coupling constant (*J_a,a'_* = 6.6 Hz) [13]. The ROESY correlations between H-a and H-6'' and between H-a' and H-6' or H-2' suggested that H-a and H-6'' were *cis* to one another; similarly, H-a' and H-6' or H-2' were *cis*. The absolute configuration of compound **3** was assigned based on the finding that the experimental data and calculated ECD spectrum for the a *R*, a' *R* configuration of compound **3** matched exactly (Figure 3).

Loddigesiinol J (compound **4**, Figure 1) has a molecular formula of C_31_H_28_O_8_ based on HRESIMS data (observed *m/z* 527.17132 [M-H]^−^, calculated 527.17114), which was two mass units more than that of compound **3**. The ^1^H- and ^13^C-NMR data for compound **4** were similar to those of compound **3** (Table 2). The most obvious difference between compounds **3** and **4** was that two sp^2^ methine group signals (δ_C_/δ_H_ 128.6/7.39 and 124.1/7.20) changed to two sp^3^ methylene group signals (δ_C_/δ_H_ 30.2/2.46 and 27.5/2.15/2.35). These results suggested that a double bond was replaced by a C-C single bond in compound **4**. The HMBC correlations between H-10 and C-1 and between H-9 and C-4b and C-8 suggested that the C-C single bond was between C-9 and C-10 (Figure S24 and Table S2, Supporting Information). The *trans* relationship of H-a and H-a' was assigned based on the coupling constant (*J_a,a'_* = 6.6 Hz) [13]. The absolute configuration of compound **4** was determined based on the result that the experimental ECD spectrum and calculated ECD spectrum for the a*S*, a' *S*-configuration of compound **4** matched exactly (Figure 3).

**Table 2:** NMR data for compounds **3**–**4**.

No.	3 *^a^*		4 *^a^*	
	*δ_H_* (*J*)	*δ_c_*	*δ_H_* (*J*)	*δ_c_*
1		115.6, C		119.5, C
2		158.7, C		159.9, C
3	6.89, s	97.9, CH	6.47, s	97.1, CH
4		158.3, C		156.2, C
4a		133.8, C		133.8, C
4b		126.2, C		126.3, C
5	9.61, d (9.0)	130.3, CH	8.22, d (9.0)	130.0, CH
6	7.15, dd (9.0, 2.4)	117.4, CH	6.67, dd (8.4, 1.8)	113.6,CH
7		155.3, C		156.0, C
8	7.16, brs	112.4, CH	6.62, brs	115.0, CH
8a		115.8, C		139.5, C
9	7.39, d (9.0)	128.6, CH	2.46, t (7.8)	30.2, CH_2_
10*α*	7.20, d (9.6)	124.1, CH	2.35, td (15.0, 7.8)	27.5, CH_2_
10*β*			2.15, dt (15.0, 7.8)	
10a		131.0, C		136.9, C
1'		133.2, C		133.0, C
2'	6.70, s	104.5, CH	6.65, s	104.5, CH
3'		148.9, C		148.9, C
4'		136.9, C		137.3, C
5'		overlap with 3'		overlap with 3'
6'	6.70, s	overlap with 2'	6.65, s	overlap with 2'
1''		136.4, C		135.8, C
2''	6.87, s	112.2, CH	6.81, s	112.1, CH
3''		148.8, C		148.7, C
4''		146.6, C		146.6, C
5''	6.79, d (8.4)	116.2, CH	6.79, d (7.8)	116.2, CH
6''	6.67, d (7.8)	121.5, CH	6.62, d (7.8)	121.5, CH
A	5.49, d (6.6)	95.3, CH	5.33, d (7.2)	94.9, CH
a'	4.86, d (6.6)	58.0, CH	4.52, d (6.6)	57.5, CH
3'-CH_3_O	3.78, s	56.8, CH_3_	3.79, s	56.8, CH_3_
3''-CH_3_O	3.72, s	56.4, CH_3_	3.76, s	56.5, CH_3_
5'-CH_3_O	overlap with 3'	overlap with 3'	overlap with 3'	overlap with 3'
7-OH	8.52, s		8.70, s	
4-OH	9.70, s		8.13, s	
4''-OH	7.57, s		7.56, s	
4'-OH	7.32, s		7.31, s	

The data were recorded at *^a^* 600 MHz (^1^H-NMR) and 150 MHz (^13^C-NMR); chemical shifts (*δ*) are in ppm and coupling constants (*J*) are in Hz.

The structure of compound **5** was also elucidated by the spectroscopic data. The *trans* relationship of H-2 and H-3 was assigned based on the coupling constant (*J_2,3_* = 8 Hz, Table S3, Supporting Information) [14,15]. Its absolute configuration was determined as 2*S*, 3*R*-configuration from the negative Cotton effect at 270 nm (*∆ε* −0.57) and positive Cotton effect at 294 nm (*∆ε* +1.5) in the ECD spectrum (Figure S23, Supporting Information), which correspond well with the ECD spectra of (2*S*,3*R*)-*trans*-flavan-3-ols in literatures [16,17,18]. It was found that the planar structure of compound **5** was the same as that of known crepidatuol B [19], but the absolute configuration of crepidatuol B has been reported.

**Figure 4:**
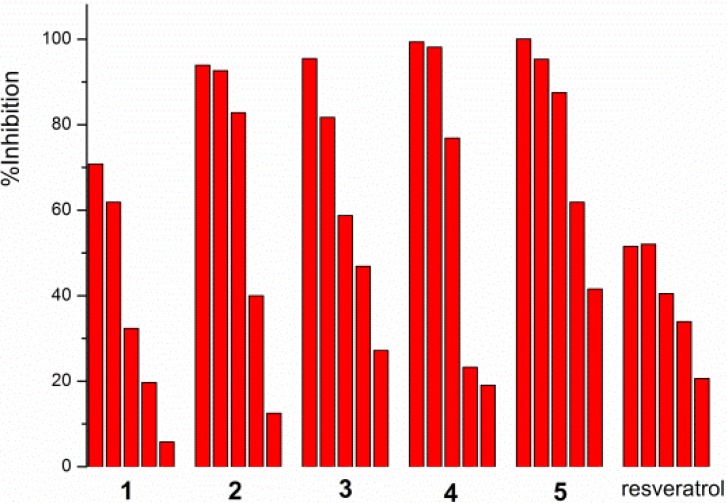
Concentration-dependent α-glucosidase inhibition of compounds **1**–**5** and resveratrol (concentration from high to low, **1** and **2**: 0.020, 0.018, 0.015, 0.010, 0.005 mM; **3** and **4**: 0.015, 0.010, 0.0050, 0.0015, 0.0010 mM; **5**: 0.08, 0.050, 0.030, 0.020, 0.018 mM; resveratrol: 0.050, 0.030, 0.020, 0.016, 0.010 mM).

Compounds **1**–**5** were evaluated *in vitro* for the *α*-glucosidase inhibitory activity by the chromogenic method using p-nitrophenyl-*α*-D-glucopyranoside as substrate [20]. The bioassay showed that compounds **1**–**5** had strong inhibit activities against *α*-glucosidase with IC_50_ values of 16.7, 10.9, 2.7, 3.2 and 18.9 μM, respectively (Table 3), and the activities were in a concentration-dependent manner (Figure 4). *Trans*-resveratrol was used as a positive control, which is a well-known naturally occurring hydroxystilbene, and it has been reported that *trans*-resveratrol has a more potent inhibitory effect than the clinical drug acarbose [11,12]. Our test results indicated that compounds **1**–**5** were significantly stronger than *trans*-resveratrol. Loddigesiinols I (**3**) and J (**4**) were 10-fold more potent *α*-glucosidase inhibitors than *trans*-resveratrol.

**Table 3:** Inhibitory effects of compounds **1**–**5** against α-glucosidase (*n* = 3).

Compound	1	2	3	4	5	*trans*-Resveratrol *^c^*
IC_50_ (μΜ)	16.7	10.9	2.7	3.2	18.9	27.9

*^c^* Positive control.

## 3. Experimental

### 3.1. General Information

*α*-Glucosidase, its substrate 4-nitrophenyl-*α*-D-glucopyranoside and dimethyl sulfoxide were purchased from Sigma (St. Louis, MO, USA). *Trans*-resveratrol was purchased from National Institute for the Control of Pharmaceutical and Biological Products (Beijing, China). Methanol was HPLC grade. Other reagents were analytical grade and commercially available. Optical rotation measurements were carried out using a Bellingham+Stanley 37–440 polarimeter (Bellingham & Stanley Ltd., Kent, UK). UV spectra were determined using a UV-240 spectrophotometer (Shimadzu, Tokyo, Japan). ECD spectra were measured using a Jasco J-810 Circular Dichroism Spectrometer (JASCO Corporation, Tokyo, Japan). IR spectra were measured on a TENSOR37 (Bruker Optics, Ettlingen, German) spectrometer. The ^1^H-NMR and ^13^C-NMR data were acquired using a Bruker Avance 400 spectrometer at 400 MHz for ^1^H nuclei and 100 MHz for ^13^C nuclei, a Bruker Avance III 500 MHz NMR spectrometer at 500 MHz for ^1^H nuclei and 125 MHz for ^13^C nuclei, and a Bruker Avance III 600 MHz NMR spectrometer at 600 MHz for ^1^H nuclei and 150 MHz for ^13^C nuclei (Bruker Biospin, Rheinstetten, German). TMS was used as an internal standard, and the chemical shifts (*δ*) were expressed in ppm. The ESI Mass spectra were obtained using LCQ-DECA-XP (Thermo) liquid chromatography-mass spectrometry (LC-MS). High-resolution mass spectra were measured on a LTQ-Orbitrap LC-MS (Thermo Fisher, Frankfurt, German). HPLC was performed using a 515 pump with a UV 2487 detector (Waters, Milford, USA) and an Ultimate XB-C-18 column (250 × 10 mm, 5 μL; Welch, MD, USA). Normal pressure preparative column chromatography was carried out on RP-18 gel (25–40 μm, Daiso Inc., Osaka, Japan), silicagel (200–400 mesh, Qingdao Marine Chemical Inc., Qingdao, China), or Sephadex-LH-20 (GE Healthcare, Stockholm, Sweden) for reversed and direct phase elution modes, respectively. TLC was performed over F_254_ glass plates (Qingdao Marine Chemical Inc.) and analyzed under UV light (254 and 366 nm).

### 3.2. Plant Material

The stems of *D. loddigesii* (from Yunnan Province, China) were purchased in September 2011 from CAIZILIN pharmacy of Guangzhou, China and identified with classical method by Pharmaceutical botanist Prof. Lin Jiang, School of Pharmaceutical Sciences, Sun Yat-sen University. A voucher specimen (No. 20110925) has been deposited in the School of Pharmaceutical Sciences, Sun Yat-sen University, Guangzhou.

### 3.3. Extraction and Isolation

The air-dried stems of *D. loddigesii* (4 kg) were macerated with methanol (35 L twice for 7 days each) at room temperature to generate 110 g of crude extract. The crude extract was suspended in H_2_O (2 L) and partitioned with *n*-hexane (2 L × 3), EtOAc (2 L × 3), and *n*-BuOH (2 L × 3) to give *n*-hexane (13 g), EtOAc (30 g), *n*-BuOH (47 g), and H_2_O (16 g) extracts, respectively. The EtOAc extract was purified by Sephadex LH-20 (3.5 × 100 cm) eluted with MeOH to give 30 fractions. Fractions 20–30 were combined and evaporated to dryness to give 250 mg of red extract. The red extract was then chromatographed over a column of RP-18 gel (2.5 × 30 cm, MeOH/H_2_O, 100/0 to 40/60 v/v) to yield six fractions. Fraction 1 (20 mg) was repeatedly applied to a Sephadex LH-20 column and eluted with MeOH to isolate compound **5** (7 mg). Fraction 2 (30 mg) was separated by HPLC (MeOH/H_2_O = 40/60, 2 ml/min, 254 nm) and then purified using a Sephadex LH-20 column (MeOH) to isolate compounds **1** (6 mg) and **4** (2 mg). Fraction 3 (30 mg) was separated by HPLC (MeOH/H_2_O = 55/45) to yield compound **3** (3.4 mg). Fraction 4 (30 mg) was subjected to column chromatography over silica gel (1 × 5 cm, CH_2_Cl_2_/MeOH = 100/6) and Sephadex LH-20 (MeOH) to yield compound **2** (8.8 mg).

### 3.4. Spectral Data

*Loddigesiinol G* (**1**): red amorphous solid; [a]^25^_D_ +41 (*c* 16.5, MeOH); UV (MeOH) *λ*_max_ (log*ε*) 305 (4.14), 338 (3.77), 360 (3.65) nm; ECD (CH_3_CN) *∆ε*_309_ +5.5; IR (KBr) *ν*_max_ 3446, 3027, 2925, 2858, 1738, 1635, 1455, 1434, 1382, 1366, 1229, 1216, 1110 cm^−1^; for ^1^H-NMR and ^13^C-NMR data, see Table 1, Table S1 and Table S2; ESIMS *m/z* 541 [M-H]^−^; HRESIMS *m/z* 541.15024 [M-H]^−^ (calculated for C_31_H_25_O_9_, 541.15041).

*Loddigesiinol H* (**2**): red amorphous solid; [a]^25^_D_ +82 (*c* 0.29, MeOH); UV (MeOH) *λ*_max_ (log*ε*) 280 (4.07), 307 (4.16), 360 (3.47) nm; ECD (CH_3_CN) *∆ε*_271_ −0.69, *∆ε*_308_ +5.4; IR (KBr) *ν*_max_ 3392, 3015, 2970, 2964, 1738, 1447, 1441, 1366, 1216, 515 cm^−1^; for ^1^H-NMR and ^13^C-NMR data, see Table 1, Table S1 and Table S2; ESIMS *m/z* 557 [M-H]^−^; HRESIMS *m*/*z* 557.14544 [M-H]^−^ (calculated for C_31_H_25_O_10_, 557.14532).

*Loddigesiinol I* (**3**): purplish-red amorphous solid; [a]^25^_D_ +66 (*c* 1.3, MeOH); UV (MeOH) *λ*_max_ (log*ε*) 265 (4.52), 313 (3.94), 386 (3.64) nm; ECD (CH_3_CN) *∆ε*_299_ +5.1; IR (KBr) *ν*_max_ 3436, 2968, 2925, 2863, 1616, 1517, 1482, 1310, 1205, 1112 cm^−1^; for ^1^H-NMR and ^13^C-NMR data, see Table 2, Table S1 and Table S2; ESIMS *m/z* 525 [M-H]^−^; HREIMS *m/z* 525.15528 [M-H]^−^ (calculated for C_31_H_25_O_8_, 525.15549).

*Loddigesiinol J* (**4**): pink amorphous powder; [a]^25^D +64 (*c* 0.5, MeOH); UV (MeOH) *λ*_max_ (log*ε*) 280 (4.53) nm; ECD (CH_3_CN) *∆ε*_316_ +15.7; for ^1^H-NMR and ^13^C-NMR data, see Table 2, Table S1 and Table S2; ESIMS *m/z* 527 [M-H]^−^; HRESIMS *m/z* 527.17132 [M-H]^−^ (calculated for C_31_H_27_O_8_, 527.17114).

*Crepidatuol B* (**5**): pale yellow amorphous powder; [a]^25^_D_ +6.5 (*c* 1.7, CH_3_CN); UV (MeOH) *λ*_max_ (log*ε*) 280 (3.90) nm; ECD (CH_3_CN) *∆ε*_270_ −0.57, *∆ε*_291_ +1.5; IR (KBr) *ν*_max_ 3446, 2933, 2850, 1699, 1614, 1516, 1457, 1430, 1271, 1233, 1141, 1033, and 845 cm^−1^; for ^1^H-NMR and ^13^C-NMR data, see Table S3 (Supporting Information); ESIMS *m/z* 451[M-H]^−^; HRESIMS *m/z* 451.17616 [M-H]^−^ (calculated for C_26_H_27_O_7_, 451.17623).

### 3.5. Computational Analyses

All the theoretical methods and the basis set used for optimization and spectrum calculation were recommended in previous studies [21,22]. All the theoretical calculations, including geometry optimization, frequency analysis, and ECD spectrum prediction, were carried out with the density functional theory (DFT) and time-dependent density functional theory (TDDFT) methods in the Gaussian 09 software package [23]. The geometry optimizations were performed at the B3LYP/6-31+G (d) level in the gas phase. Based on the final optimized structure, the ECD spectra were calculated at the PBE1PBE-SCRF/6-311++g (d, p) level using the PCM solvent continuum models with acetonitrile as a solvent. The theoretical predicted ECD spectra were fitted in the SpecDis software package.

### 3.6. α-Glucosidase Inhibition Assay

*α*-Glucosidase assays were performed according to referenced procedures [20]. *tran*s-Resveratrol was used as positive control and 4-nitrophenyl-*α*-D-glucopyranoside (PNPG) was used as substrate. *α*-Glucosidase (2.0 units/mL) and PNPG substrate (1 mM) were dissolved in 50 mM phosphate buffer at pH 7.0 separately. Compounds **1**−**5** (10 µmol/mL) and *tran*s-resveratrol (10 µmol/mL) were dissolved in dimethyl sulfoxide (DMSO). The solvent DMSO was used as blank. *α*-Glucosidase activity was assayed using 50 mM phosphate buffer at pH 7.0. 20 µL of tested materials at the designated concentrations and 10 µL of enzyme solution were added to 950 µL of phosphate buffer, and incubated at 37 °C for 20 min; then, 20 µL of substrate was added to initiate the enzyme reaction. Product (PNP) was monitored spectrophotometrically by measuring the absorbance (λ = 400 nm). Each experiment was repeated 3 times. The data obtained from the experiments were dealt with the professional software origin 7.0.

## 4. Conclusions

The chemical study of the stems of *D. loddigesii* resulted in the isolation of four new polyphenols (compounds **1**–**4**), and a known compound (compounds **5**) based on the previous research [10]. The five compounds showed strong inhibit *α*-glucosidase activities *in vitro*; especially, the activities of compounds **3** and **4** were 10 times more potent than those of *trans*-resveratrol. This study suggested that these polyphenols were probably the active ingredients responsible for the blood glucose lowering effects of *D. loddigesii*.

## Supplementary Materials

Supplementary materials can be accessed at: http://www.mdpi.com/1420-3049/19/6/8544/s1.
