# Supplementary material for: Loddigesiinols G–J: α-Glucosidase Inhibitors from Dendrobium loddigesii
Source: Molecules. 2014 Jun 23;19(6):8544–55. doi: 10.3390/molecules19068544 (PMC6270667; doi:10.3390/molecules19068544)
Supplement: Supplementary file 1 [file molecules-19-08544-s001.pdf]

Supporting Information

Figure S1. ^{1}H-NMR for loddigesiinol G (**1**).

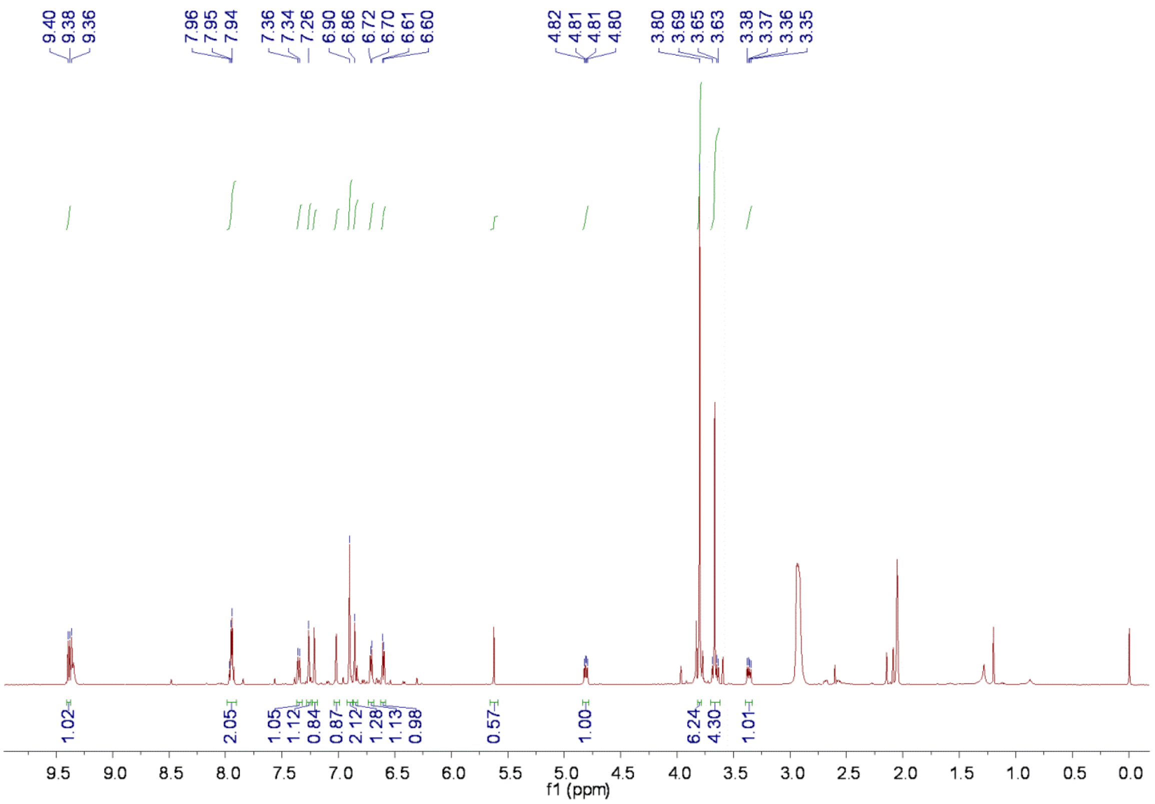

Figure S2. ^{13}C-NMR for loddigesiinol G (**1**).

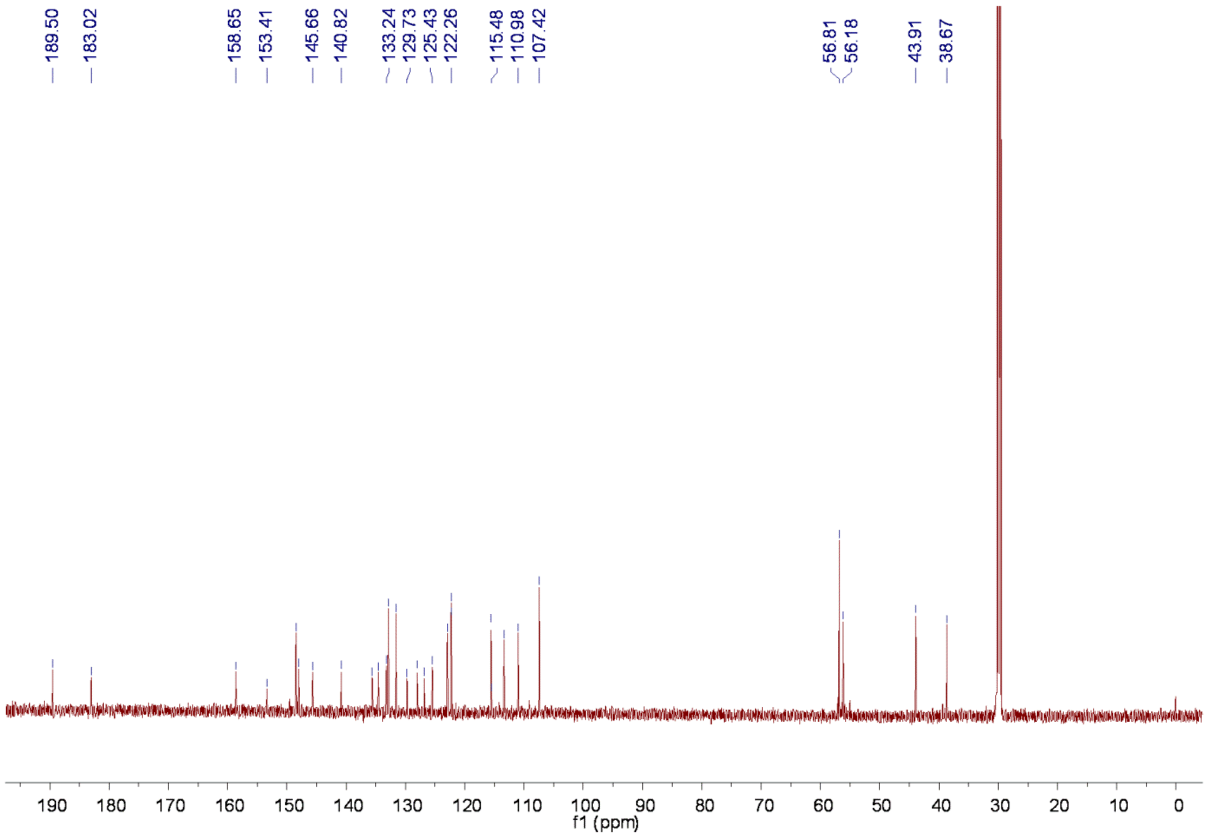

Figure S3. ^{1}H-^{1}H COSY for loddigesiinol G (**1**).

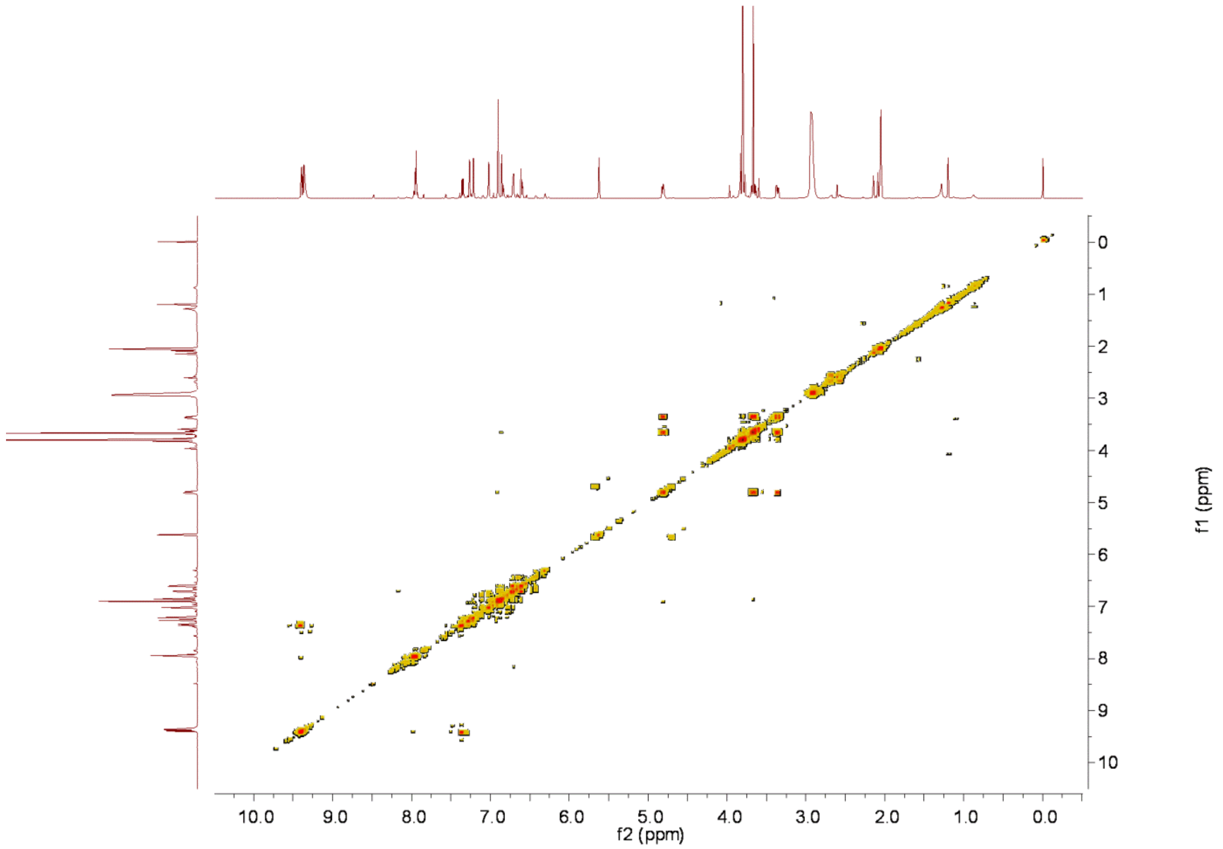

Figure S4. HSQC for loddigesiinol G (**1**).

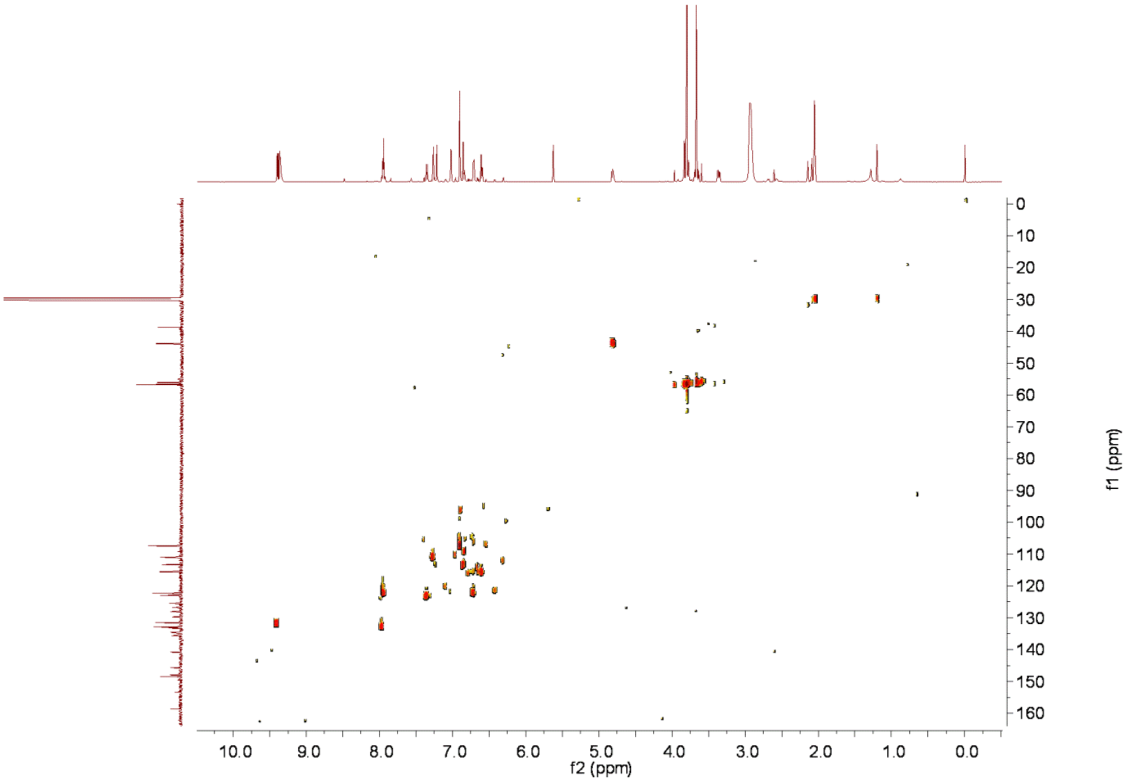

Figure S5. HMBC for loddigesiinol G (**1**).

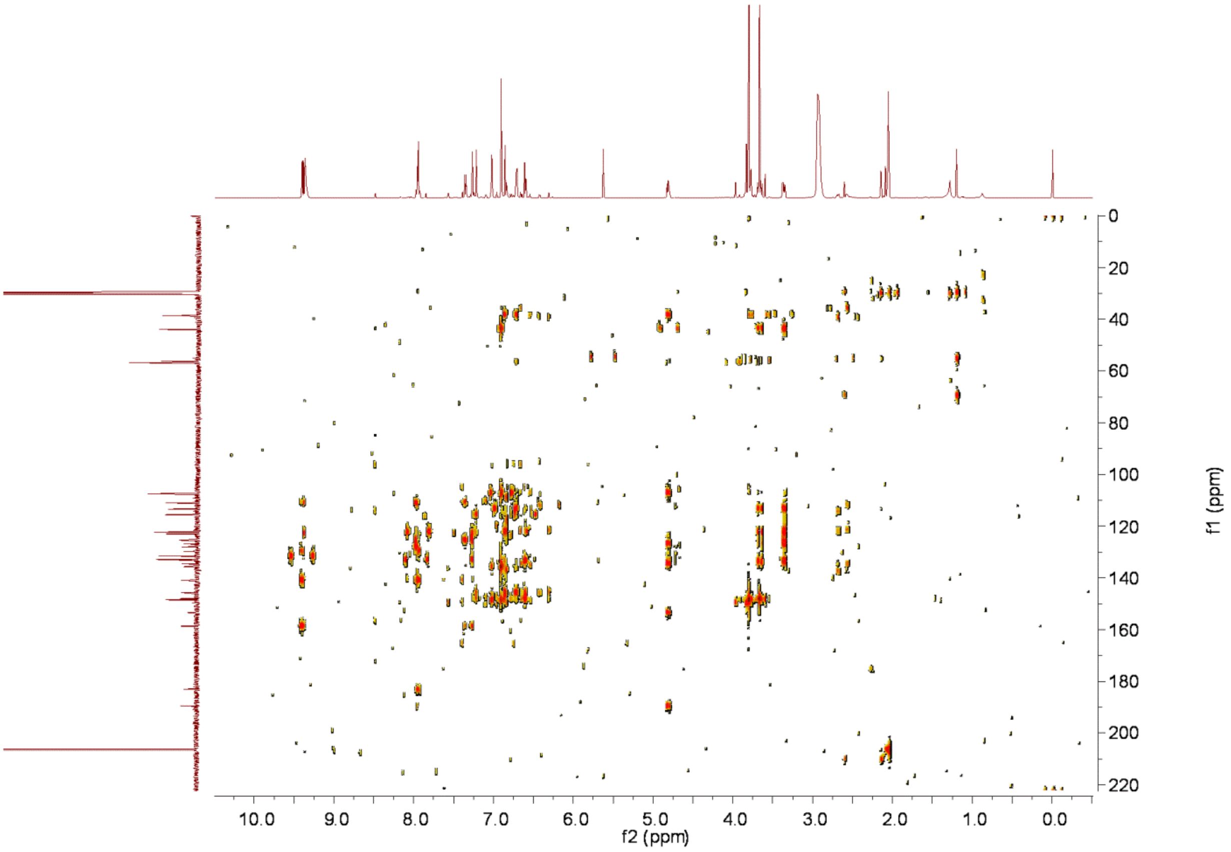

Figure S6. ^{1}H-NMR for loddigesiinol H (**2**).

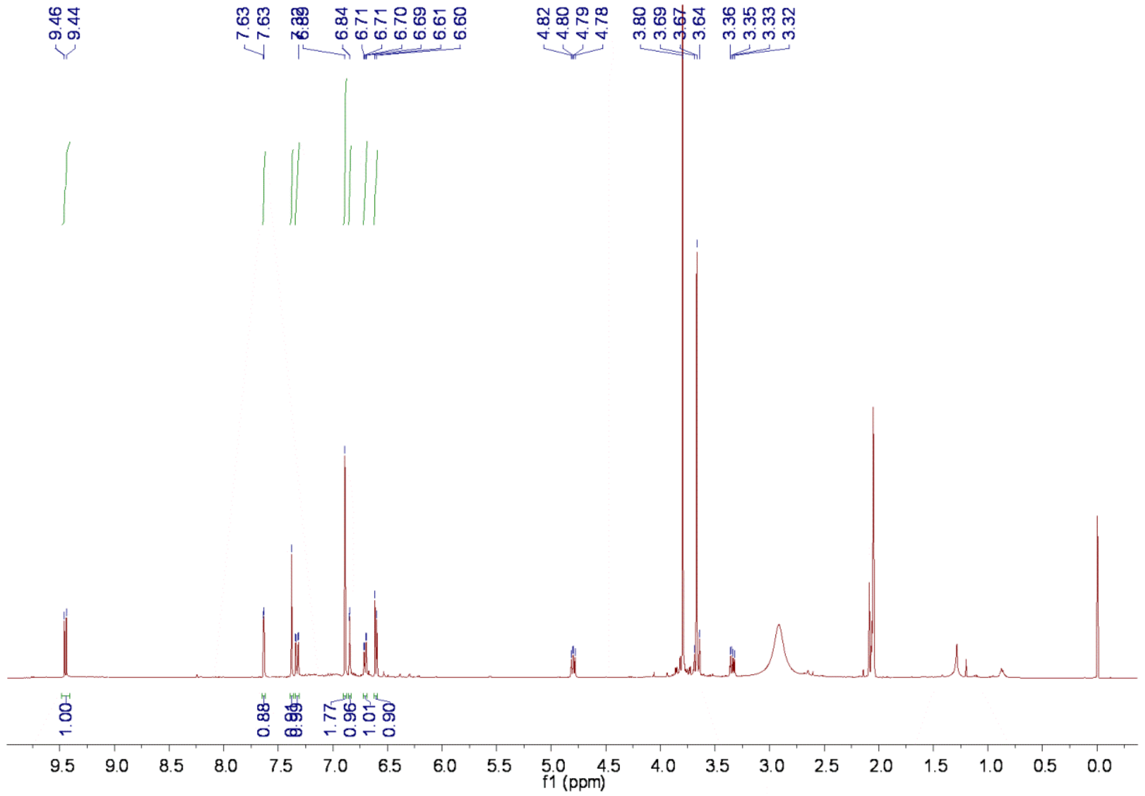

Figure S7. ^{13}C-NMR for loddigesiinol H (**2**).

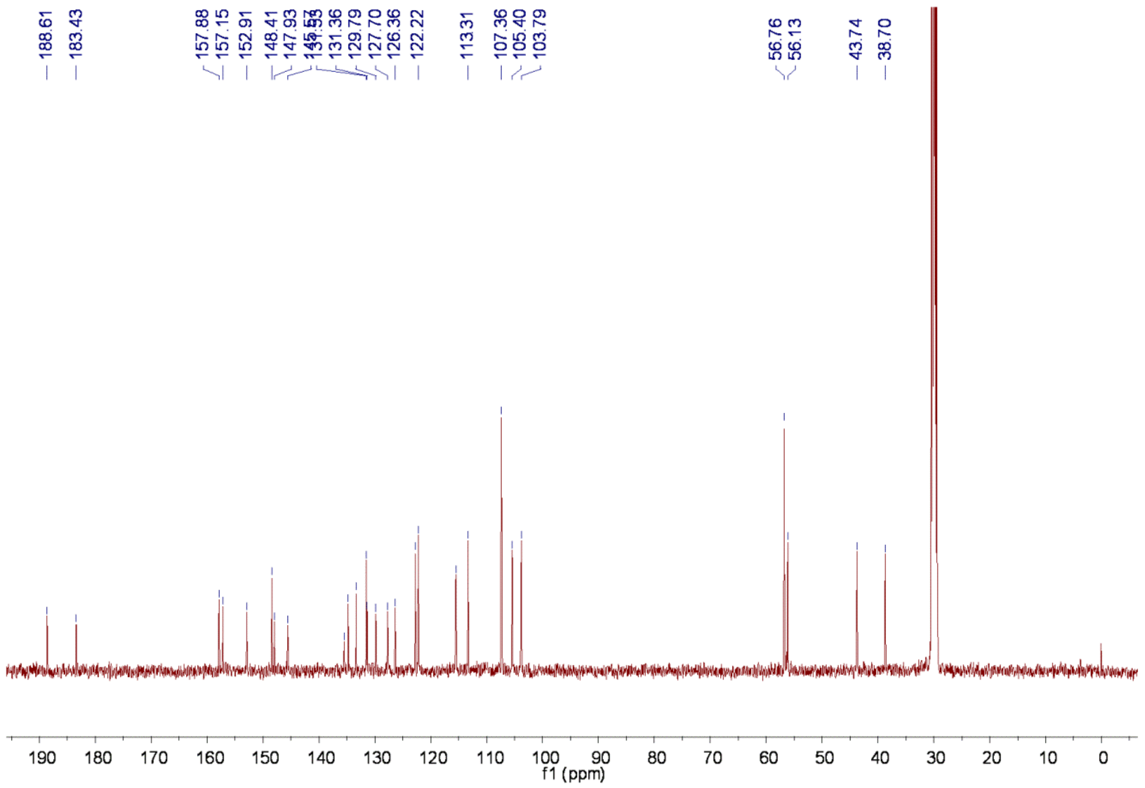

Figure S8. ^{1}H-^{1}H COSY for loddigesiinol H (**2**).

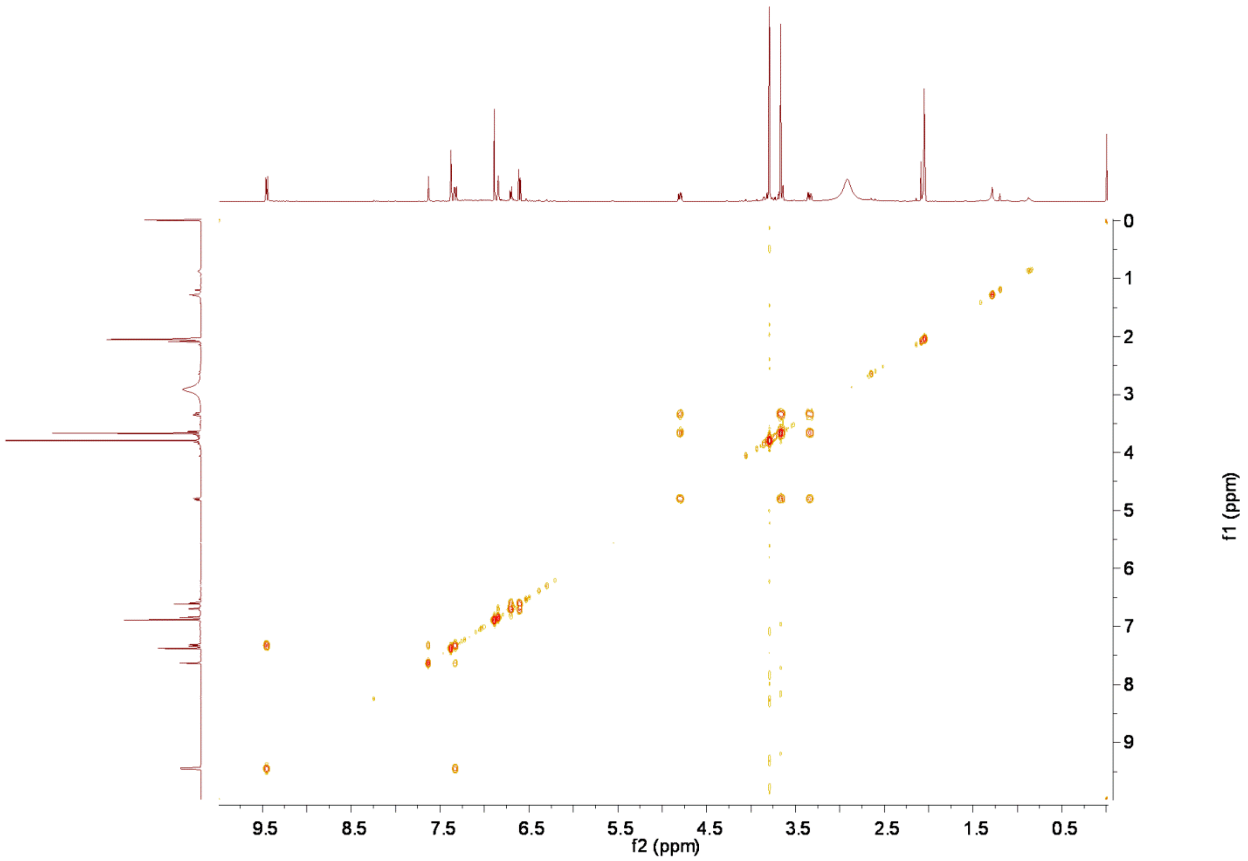

Figure S9. HSQC for loddigesiinol H (**2**).

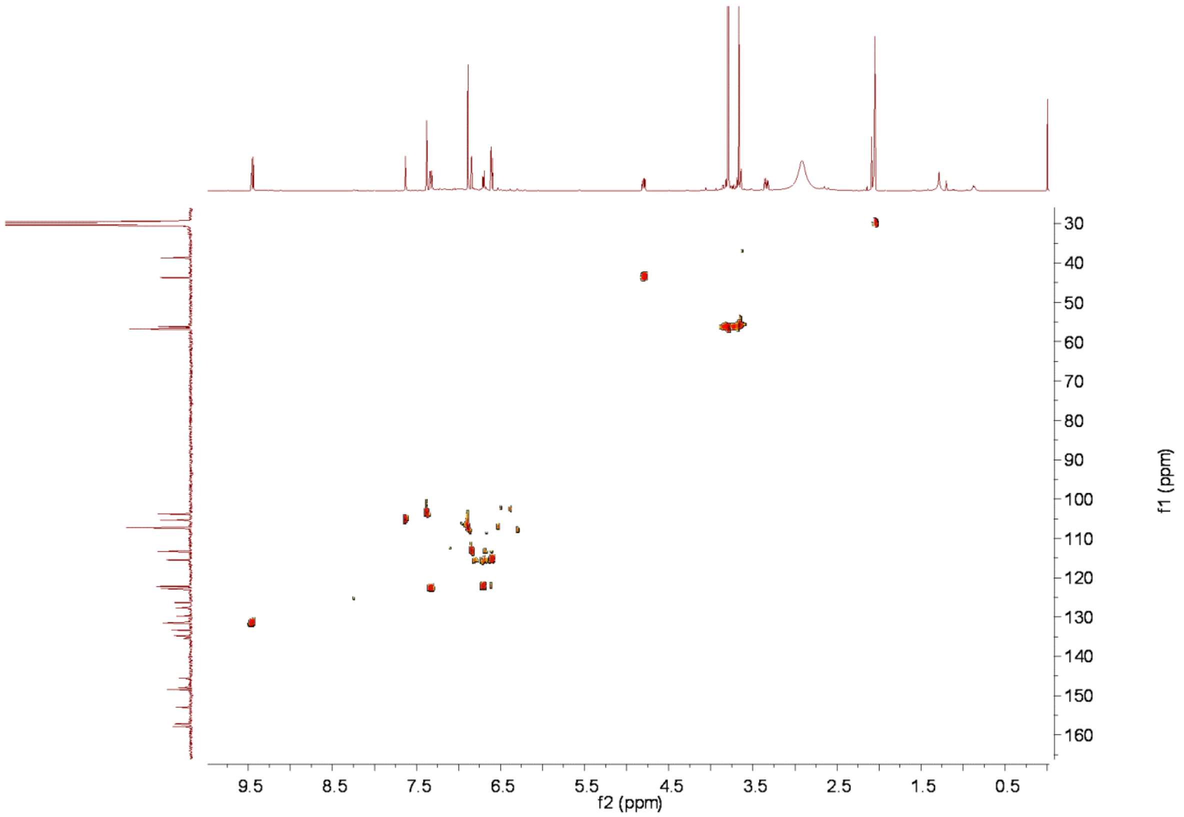

Figure S10. HMBC for loddigesiinol H (**2**).

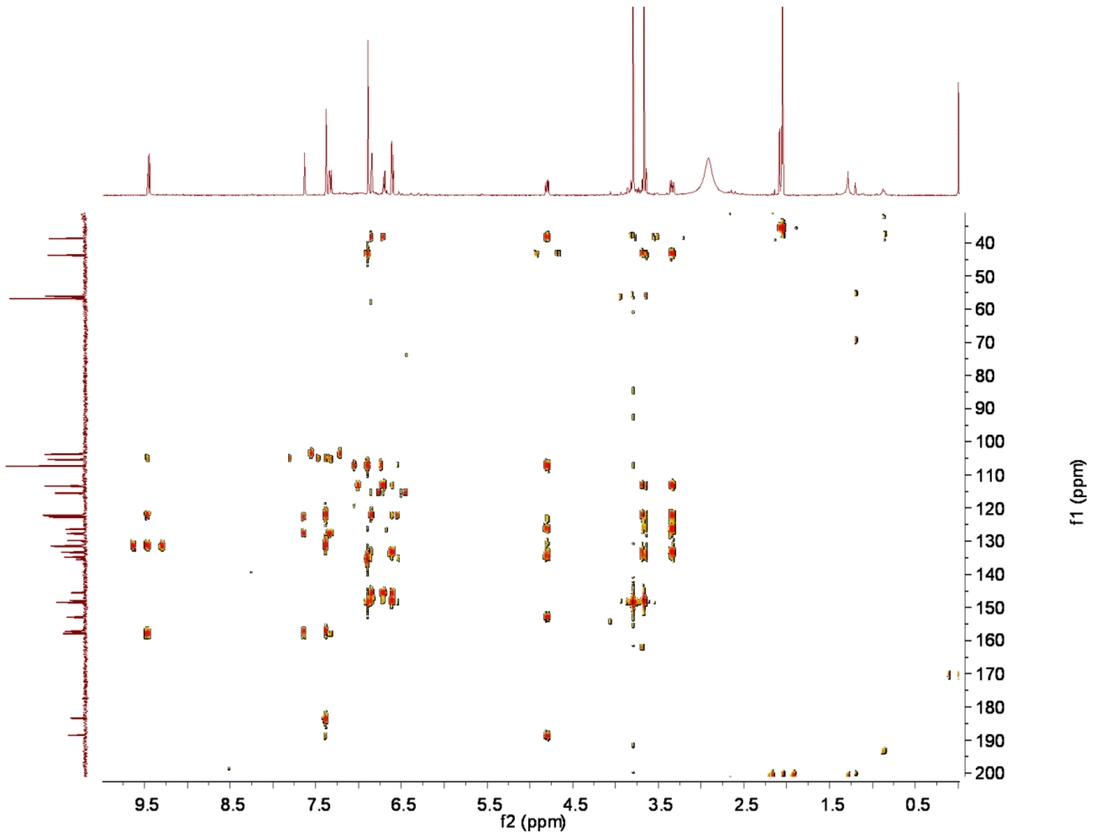

Figure S11. ECD spectrum for loddigesiinol H (**2**).

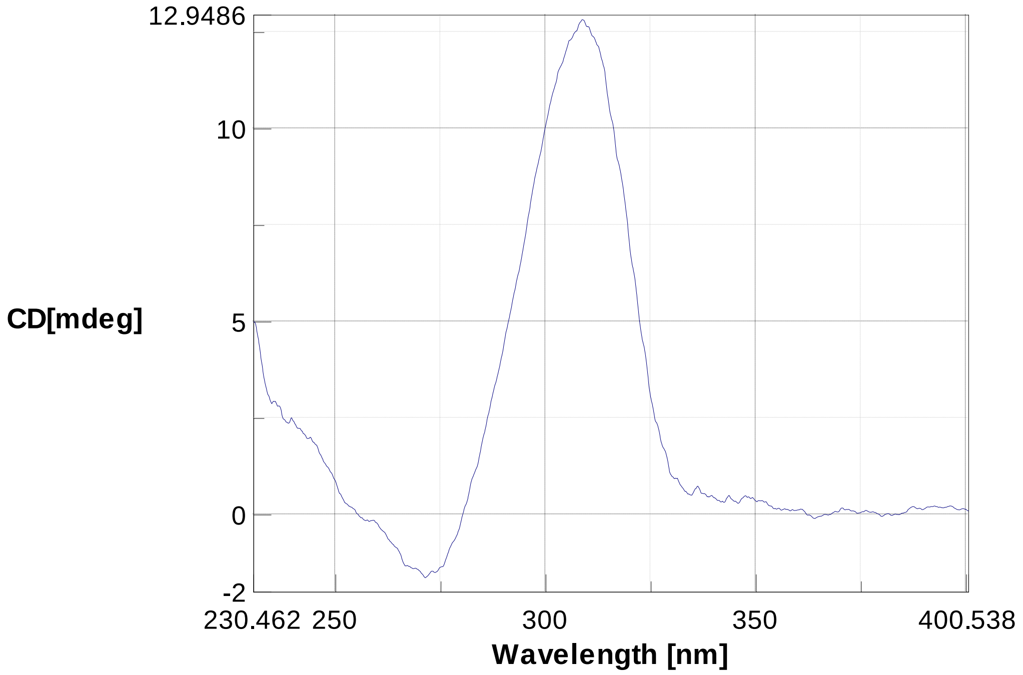

Figure S12. ^{1}H-NMR for loddigesiinol I (**3**).

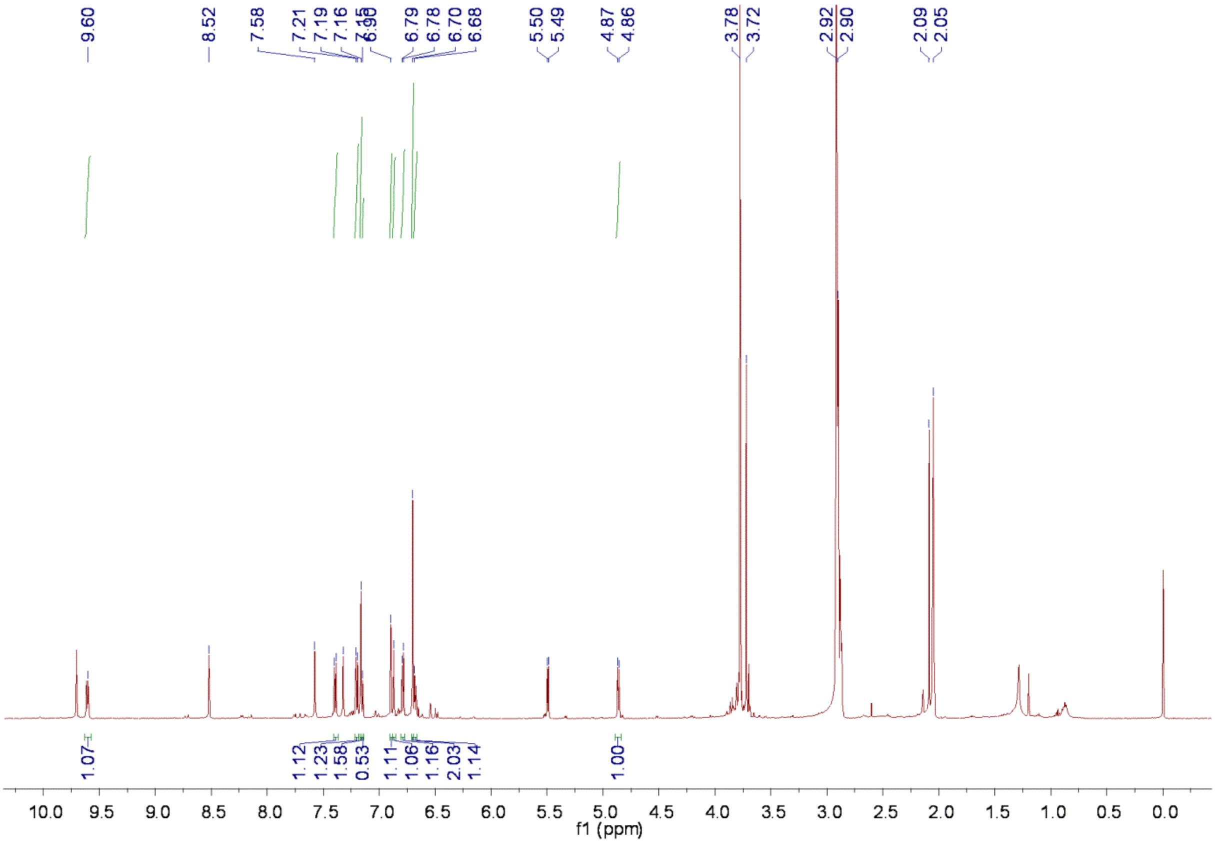

Figure S13. ^{13}C-NMR for loddigesiinol I (**3**).

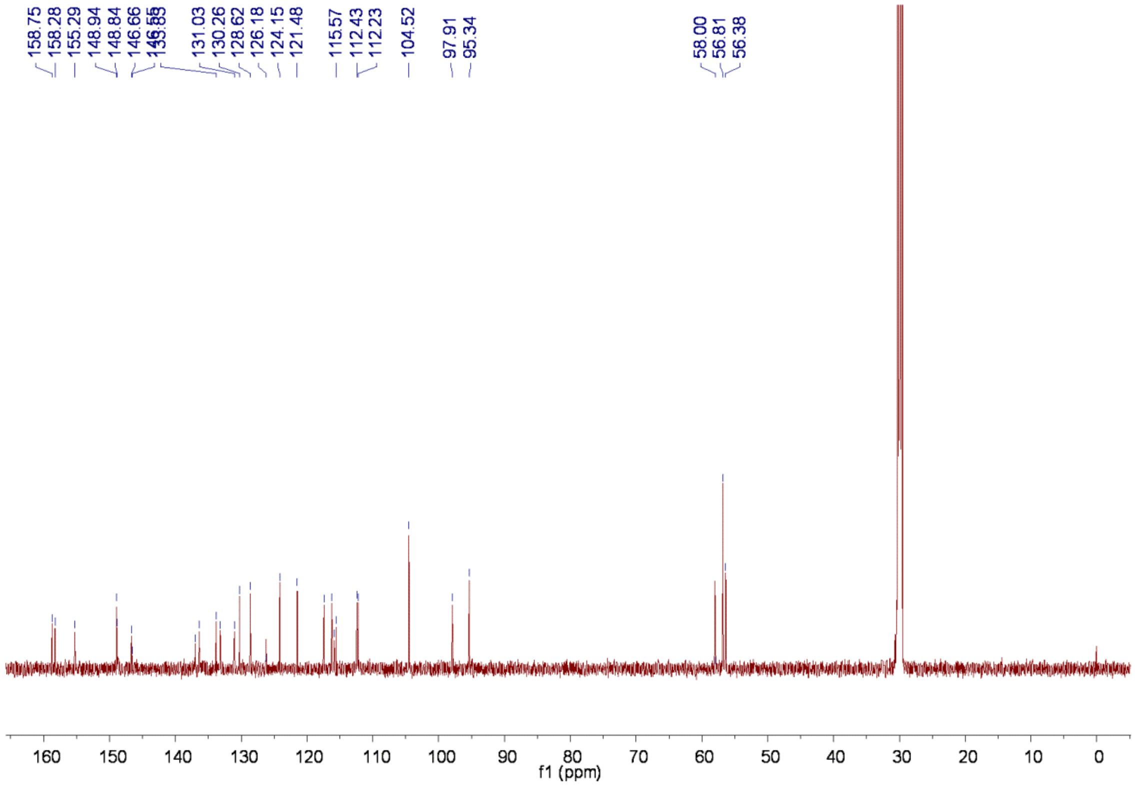

Figure S14. ^{1}H-^{1}H COSY for loddigesiinol I (**3**).

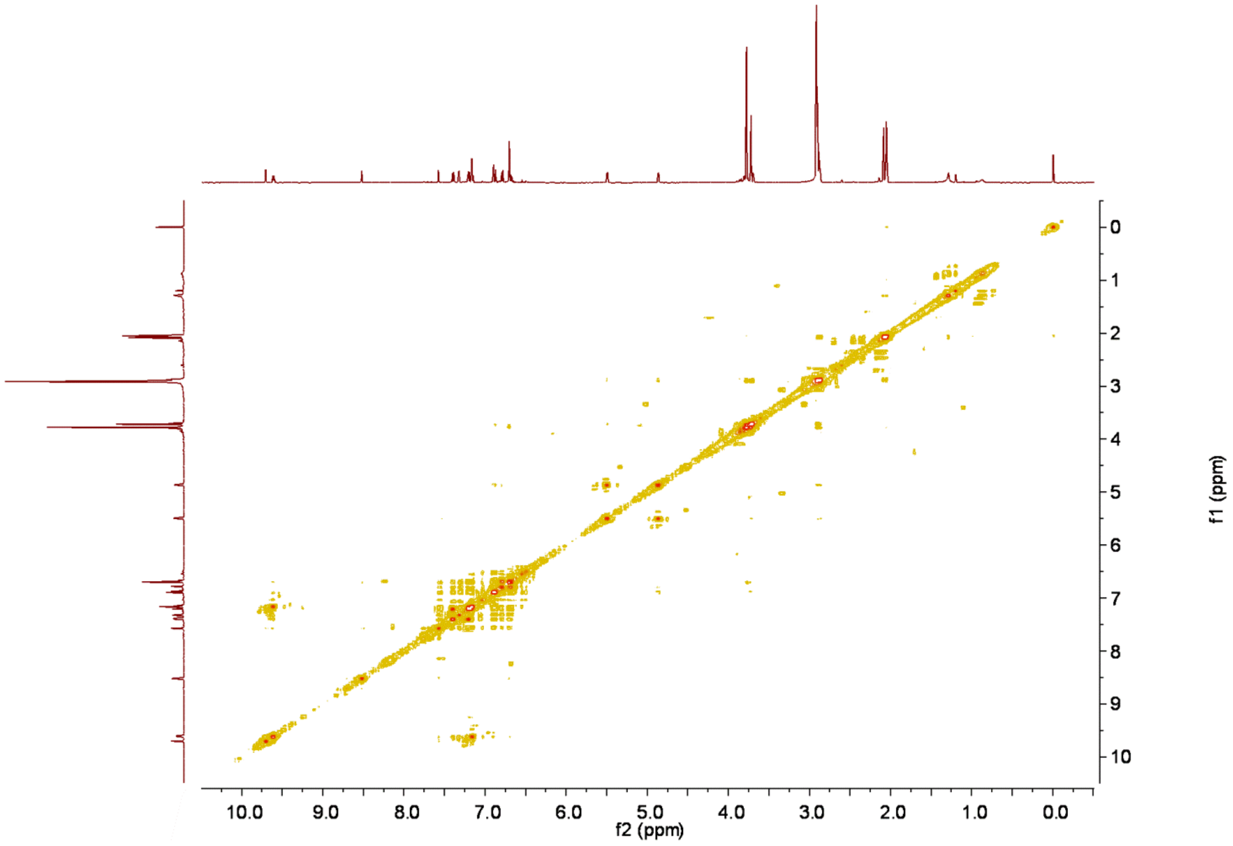

Figure S15. HSQC for loddigesiinol I (**3**).

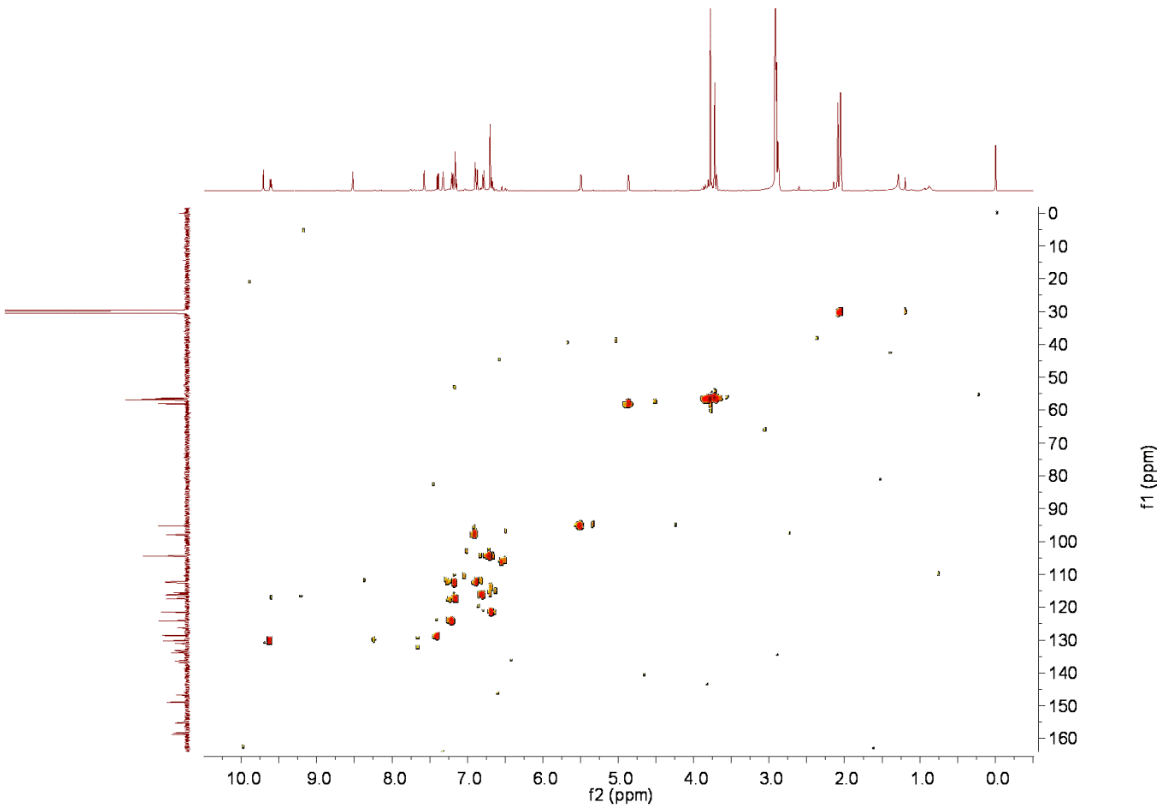

Figure S16. HMBC for loddigesiinol I (**3**).

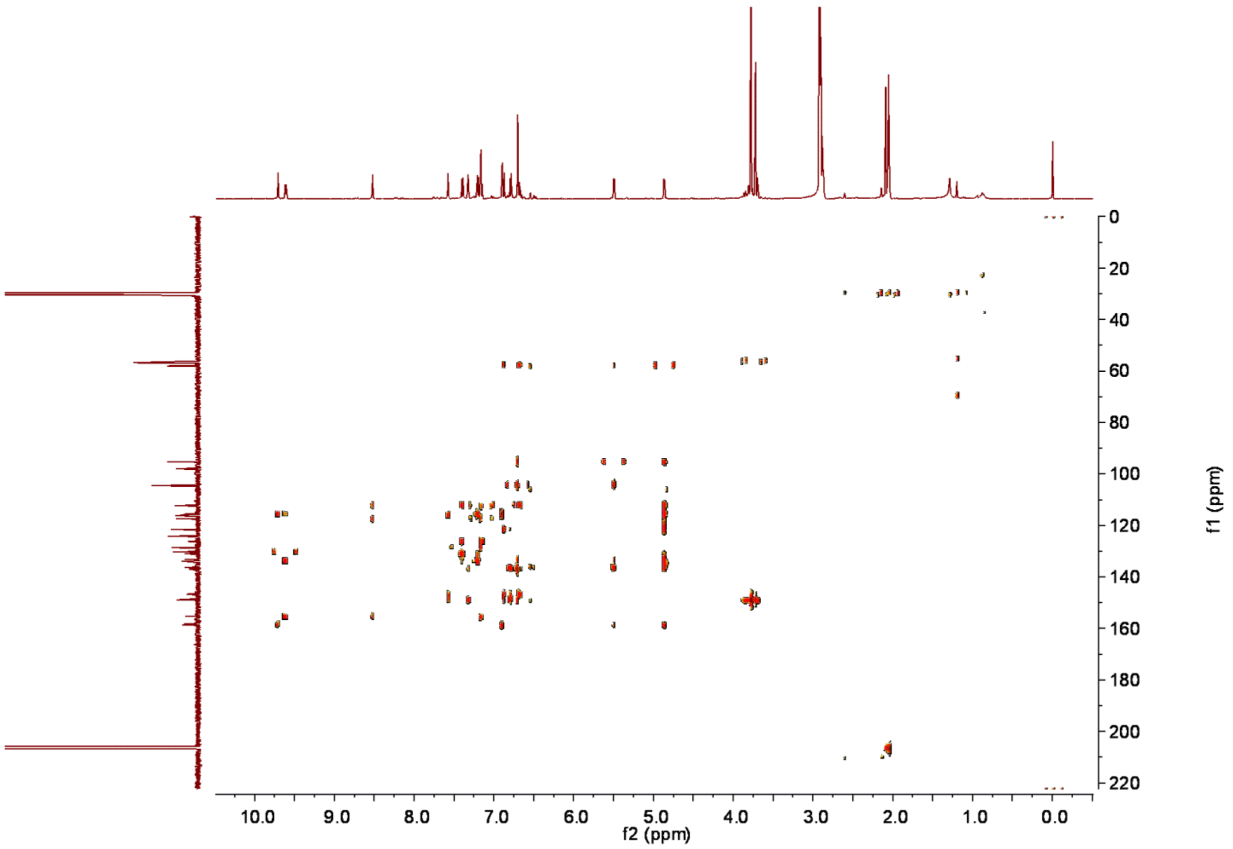

Figure S17. ROSEY for loddigesiinol I (**3**).

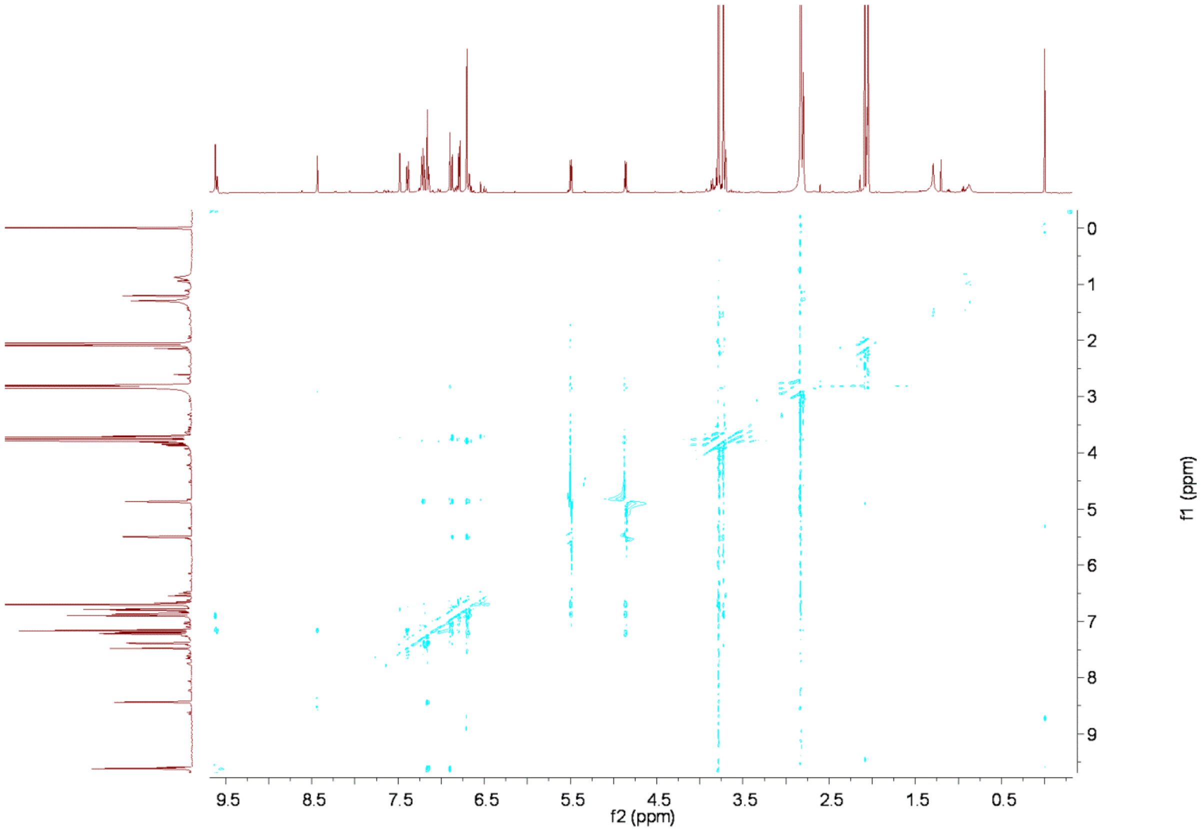

Figure S18. ^{1}H-NMR for loddigesiinol J (**4**).

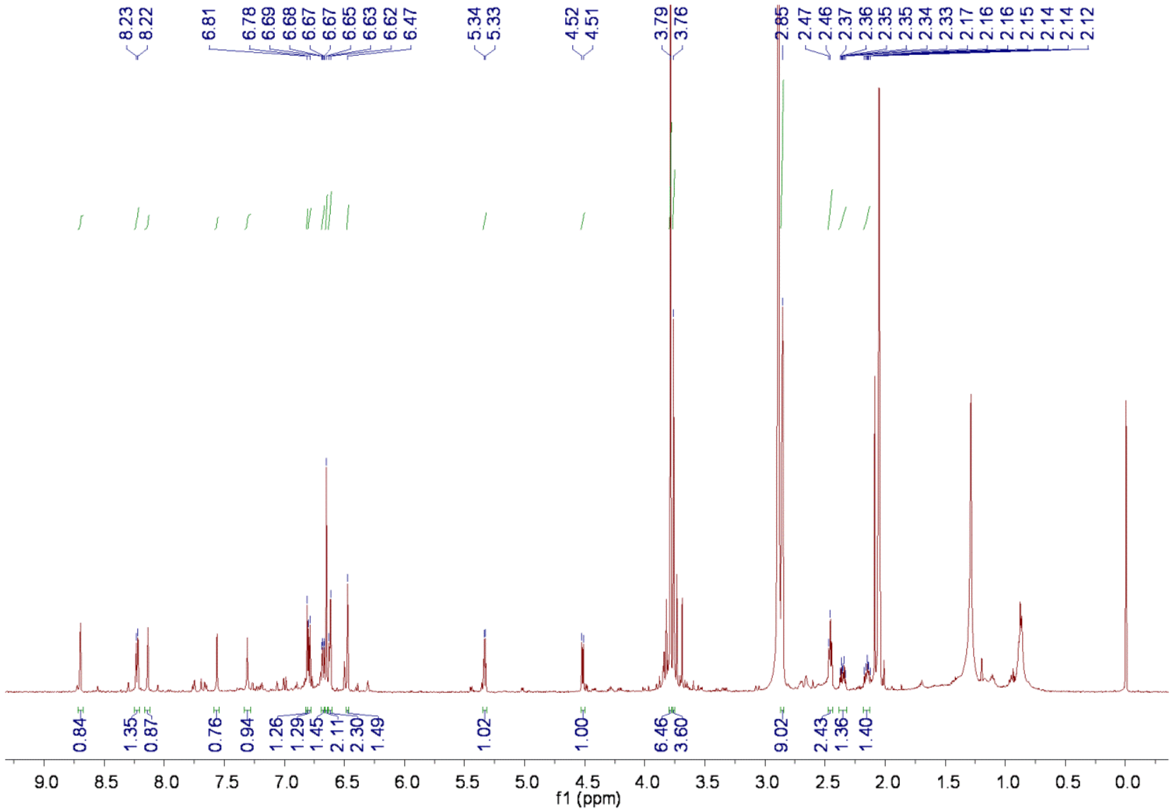

Figure S19. ^{13}C-NMR for loddigesiinol J (**4**).

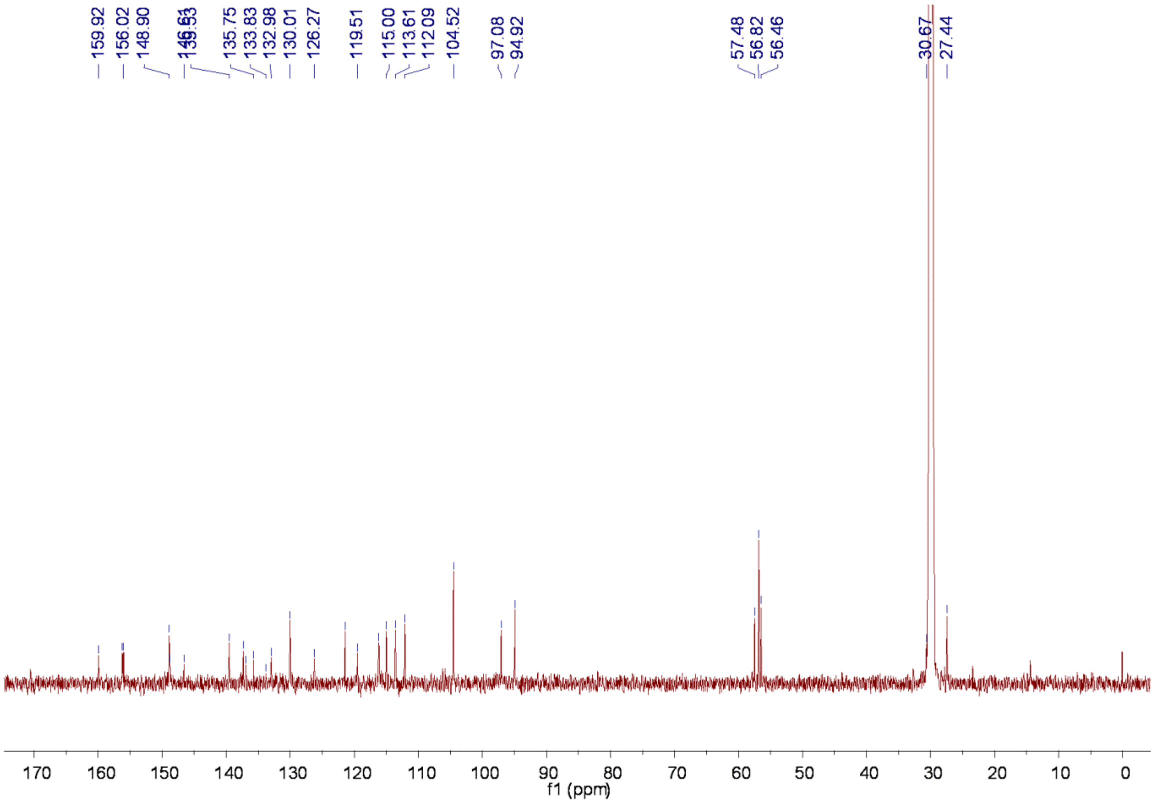

Figure S20. ^{1}H-^{1}H COSY for loddigesiinol J (**4**).

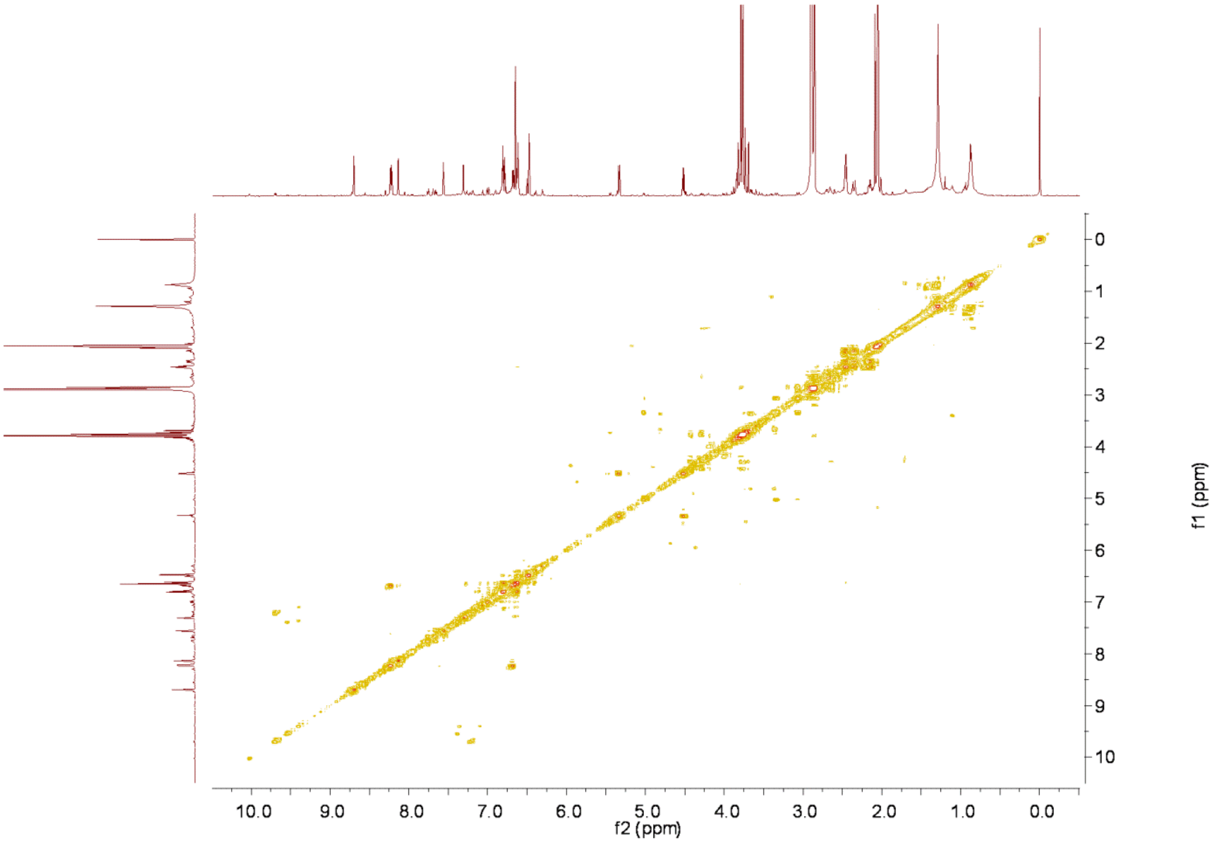

Figure S21. HSQC for loddigesiinol J (**4**).

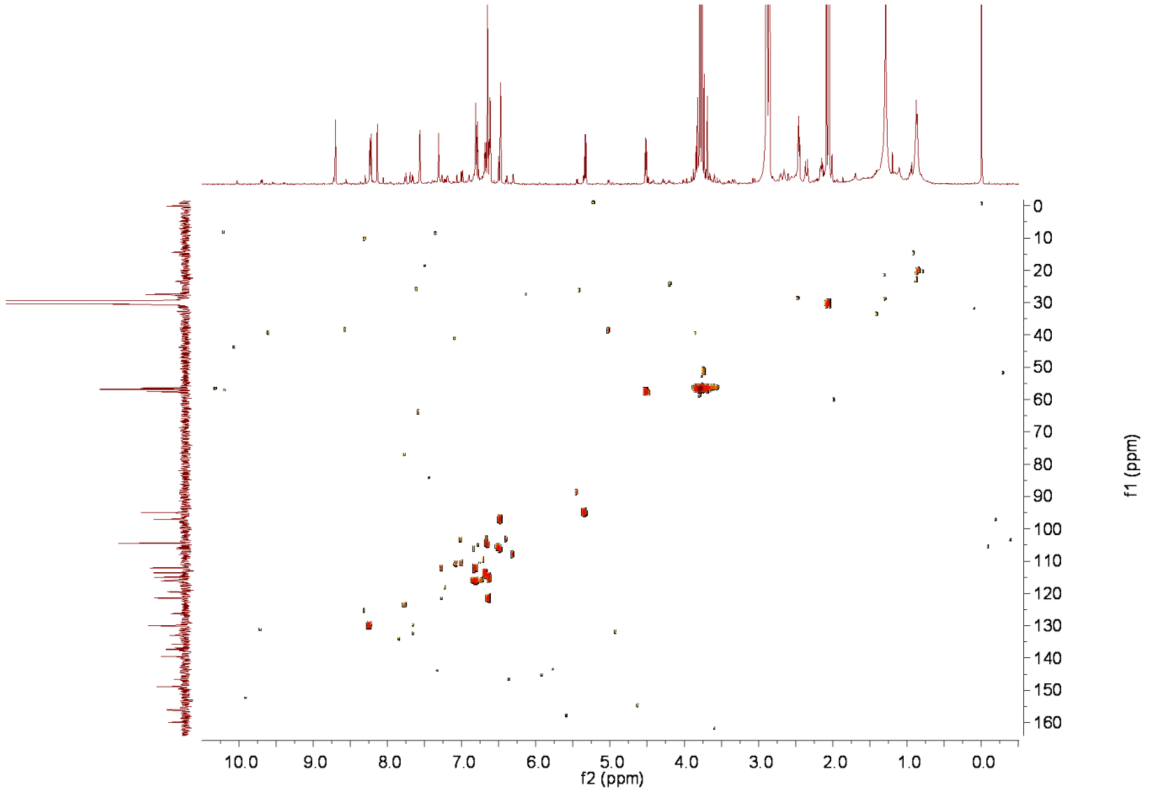

Figure S22. HMBC for loddigesiinol J (**4**).

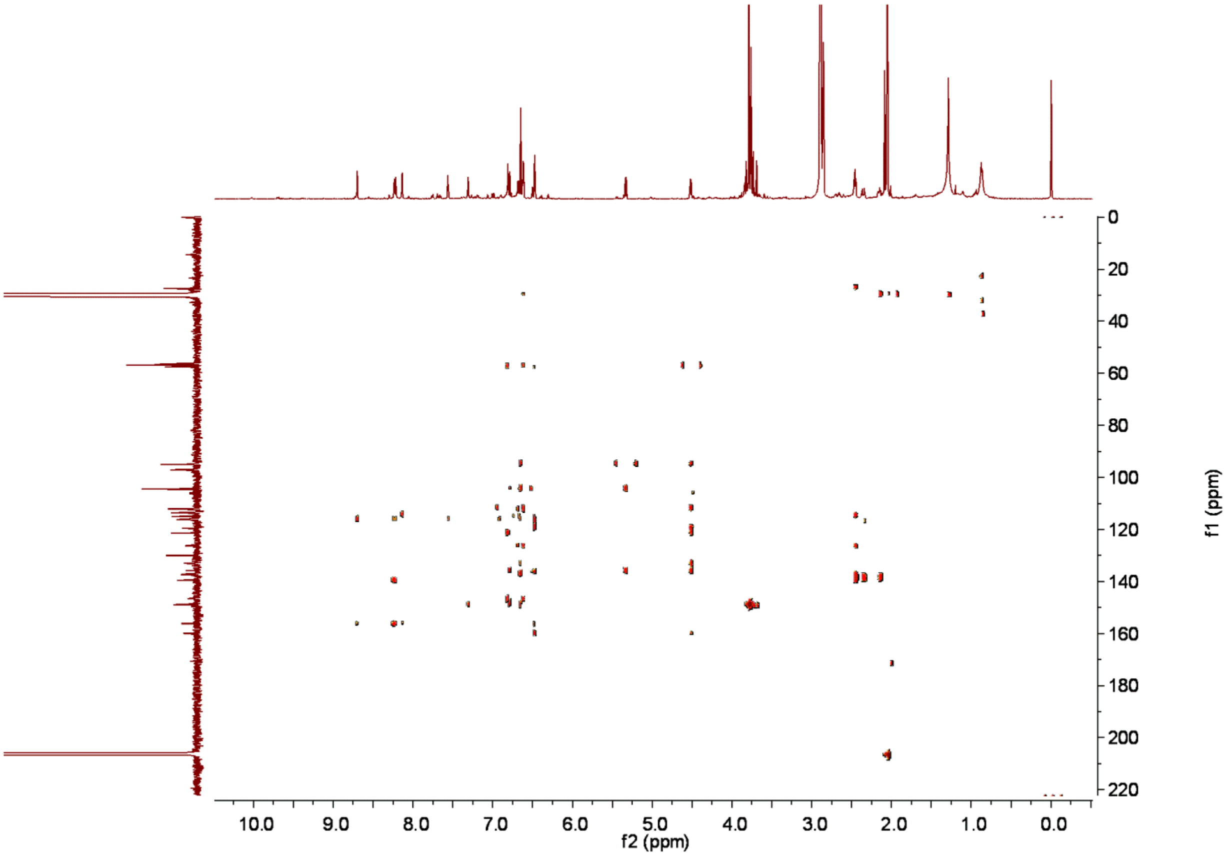

Figure S23. ECD spectrum for crepidatuol B (**5**).

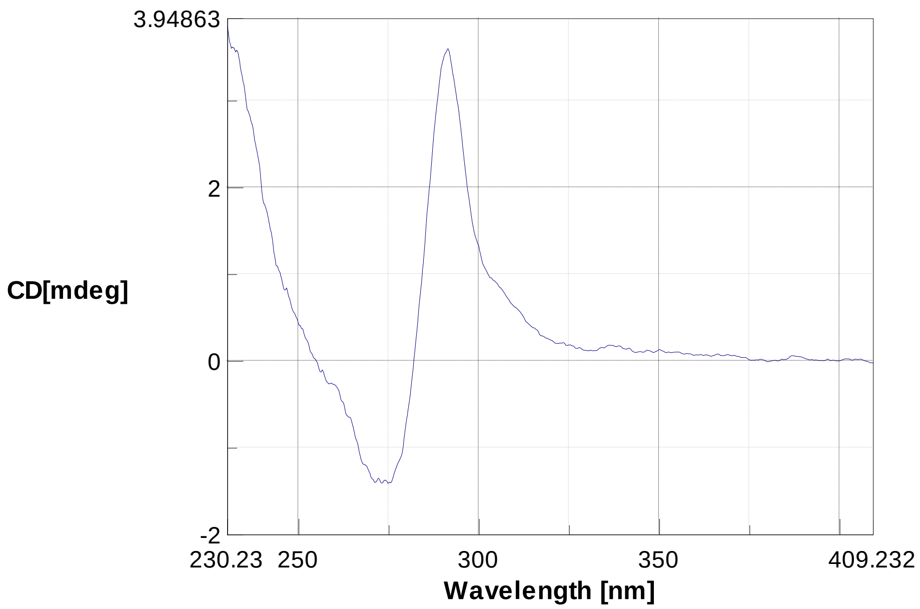

Figure S24. ^{1}H-^{1}H COSY and HMBC correlations for compounds **1**–**5**.

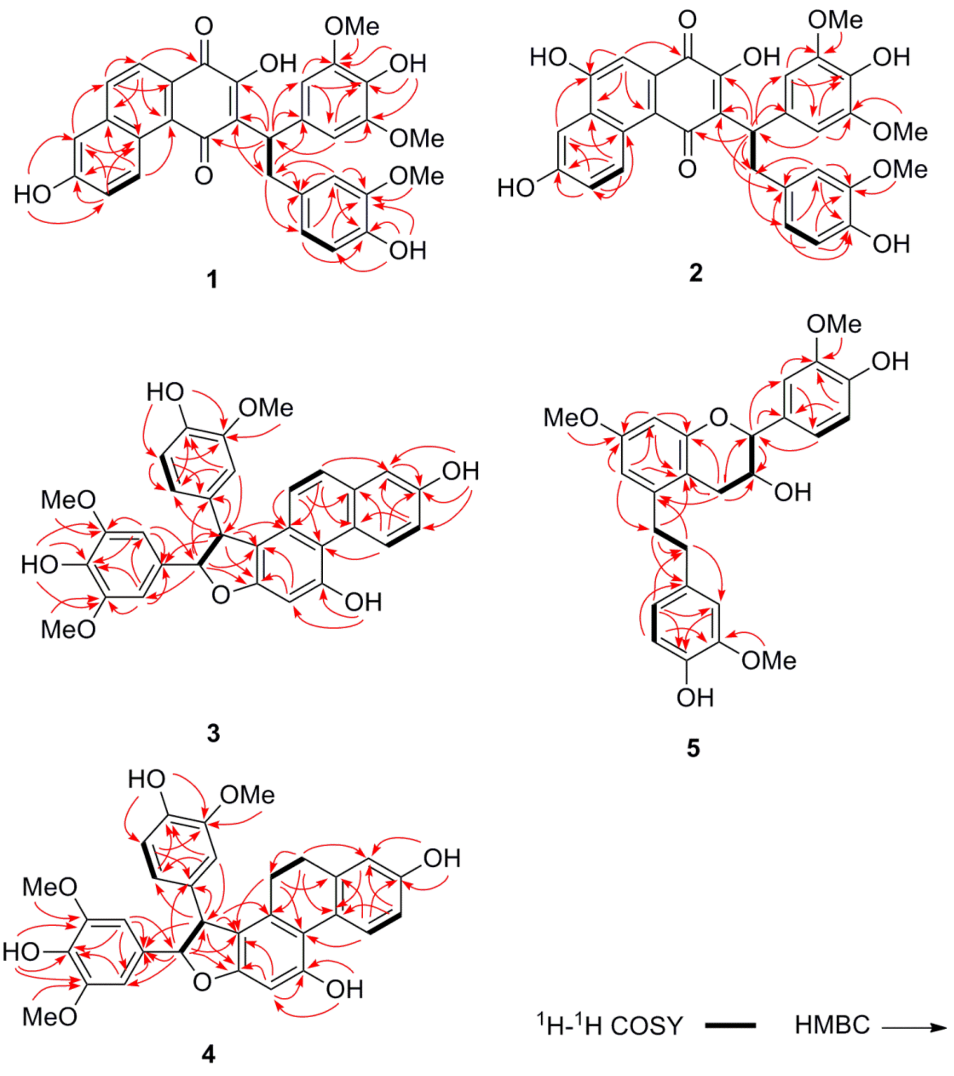

Table S1. ^{13}C Data for Compounds **1**–**4**.

	**1** [a]	**2** [b]	**3** [a]	**4** [a]
**NO.**	δ	δ	δ	δ
1	183.0, C	183.4, C	115.6, C	119.5, C
2	153.4, C	152.9, C	158.7, C	159.9, C
3	126.8, C	126.4, C	97.9, CH	97.1, CH
4	189.5, C	188.6, C	158.3, C	156.2, C
4a	140.8, C	122.1, C	133.8, C	133.8, C
4b	125.4, C	127.7, C	126.2, C	126.3, C
5	131.6, CH	131.5, CH	130.3, CH	130.0, CH
6	123, CH	122.8, CH	117.4, CH	113.6,CH
7	158.7, C	157.9, C	155.3, C	156.0, C
8	111, CH	105.4, CH	112.4, CH	115.0, CH
8a	129.7, C	131.4, C	115.8, C	139.5, C
9	132.9, CH	157.2, C	128.6, CH	30.2, CH_2
10	122.3, CH	103.8, CH	124.1, CH	27.5, CH_2
10a	128.1, C	129.8, C	131.0, C	136.9, C
1''	134.6, C	134.8, C	133.2, C	133.0, C
2''	107.4, CH	107.4, CH	104.5, CH	104.5, CH
3''	148.5, C	148.4, C	148.9, C	148.9, C
4''	135.6, C	135.5, C	136.9, C	137.3, C
5''	overlap with 3'	overlap with 3'	overlap with 3'	overlap with 3'
6''	overlap with 2'	overlap with 2'	overlap with 2'	overlap with 2'
a	133.2, C	133.3, C	136.4, C	135.8, C
a'	113.4, CH	113.3, CH	112.2, CH	112.1, CH
3'-CH_3O	148.0, C	147.9, C	148.8, C	148.7, C
3''-CH_3O	145.7, C	145.6, C	146.6, C	146.6, C
5'-CH_3O	115.6, CH	115.5, CH	116.2, CH	116.2, CH

The data were recorded at [a]150 MHz and [b]125 MHz; chemical shifts (δ) are in ppm.

Table S2. ^{1}H-NMR and HMBC Data for Compounds **1–4**.

	**1** [a]		**2** [b]		**3** [a]		**4** [a]	
**No.**	***δ*(*J*)**	**HMBC**	***δ* (*J*)**	**HMBC**	***δ* (*J*)**	**HMBC**	***δ* (*J*)**	**HMBC**
3					6.9, s	C-1, 2	6.47, s	C-1, 2, 4
5	9.39, d (9.6)	C-4a, 7, 8, 8a	9.45, d (9.5)	C-6, 7, 8, 8a, 4a	9.61, d (9.0)	C-8a, 4a, 7	8.22, d (9.0)	C-7, 8a
6	7.35, d (9.6)	C-4b, 7, 8	7.33, dd (9.5, 3.0)	C-4b, 7, 8	7.15, dd (9.0, 2.4)	C-4b, 7, 8	6.67, dd (8.4, 1.8)	C-4b, 8
8	7.26, brs	C-4b, 7, 9	7.63, brs	C-4b, 6, 9	7.16, brs	C-9, 7, 6	6.62, brs	C-4b, 9, 6
9	7.96, d (8.4)	C-4a, 4b, 8, 10, 10a			7.39, d (9.0)	C-4b, 8, 10a, 4a	2.46, t (7.8)	C-4b, 8, 10, 8a
10*α*	7.93, d (8.4)	C-1, 4, 4b, 4a, 8a	7.38, s	C-1, 4, 4a, 8, 8a, 9, 10a	7.20, d (9.6)	C-8a, 10a	2.35, td (15.6, 7.8)	C-1, 9, 8a
10*β*							2.15, dt (15.0, 7.8)	C-1, 9, 8a
2'	6.90, s	C-3', 4', 5', 6', a	6.89, s	C-3', 4', 5', 6', a	6.70, s	C-1', 6', 3', 4', a	6.65, s	C-1′, 6′, 3′, 4′, a
6'	6.90, s	C-2', 3', 4', 5', a	6.89, s	C-2', 3', 4', 5', a	6.70, s	C-1', 2', 3', 4', a	6.65, s	C-1′, 2′, 5′, 4′, a
2"	6.85, s	C-1", 3", 4", 6", a'	6.85, d (2.0)	C-1", 3", 4", 5", 6", a'	6.87, s	C-4", 6", a'	6.81, s	C-4″, 6″, a′
5"	6.60, d (7.8)	C-1", 3"	6.61, d (8.0)	C-1", 2", 3", 4", 6"	6.79, d (8.4)	C-1", 3", 6"	6.79, d (7.8)	C-1″, 3″
6"	6.71, d (7.8)	C-2", 4", a'	6.70, dd (8.0, 2.0)	C-2", 4", a'	6.67, d (7.8)	C-2", 4", a'	6.62, d (7.8)	C-2″, 4″, a′
a	4.81, dd (10.2, 6.6)	C-2, 3, 4, 1', 2'/6', a'	4.80, dd (10.0, 6.5)	C-2, 3, 4, 1', 2', a'	5.49, d (6.6)	C-2, 2'/6', 1", a', 1	5.33, d (7.2)	C-2, 1′, 2′/6′, 1″, a′
a'*α*	3.36, dd (13.8, 6.6)	C-1', 1", 2", 6", a	3.67, dd (13.5, 10.0)	C-3, 1", 2", 6", a	4.86, d (6.6)	C-2, 1', 1", 6", a, 10a, 1	4.52, d (6.6)	C-1, 2, 1′, 1″, 2″/6″, a, 10a
a'*β*	3.66, dd (13.8, 10.2)	C-1', 1", 2", 6", a	3.34, dd (13.5, 7.0)	C-3, 1", 2", 6", a				
3'-CH_3O	3.80, s	C-3'	3.8, s	C-3'	3.78, s	C-3', 5'	3.79, s	C-3′, 5′
3"-CH_3O	3.67, s	C-3"	3.67, s	C-3"	3.72, s	C-3"	3.76, s	C-3″

Table S2. *Cont.*

	**1** [a]		**2** [b]		**3** [a]		**4** [a]	
**No.**	**δ (*J*)**	**HMBC**	**δ (*J*)**	**HMBC**	**δ (*J*)**	**HMBC**	**δ (*J*)**	**HMBC**
5'-CH_3O	overlap with 3'	C-5'	overlap with 3'	C-5'	overlap with 3'	overlap with 3'	overlap with 3'	overlap with 3'
9-OH			unobserved					
2-OH	unobserved		unobserved					
7-OH	9.36, brs	C-6, 8	unobserved		8.52, s	C-7, 6, 8	8.70, s	C-7, 8
4-OH					9.7, s	C-4, 3	8.13, s	C-4, 3
4''-OH	7.21, brs		unobserved		7.57, s	C-3'', 5''	7.56, s	C-3″, 5″
4'-OH	7.02, brs		unobserved		7.32, s	C-3', 5', 4'	7.31, s	C-3′, 5′, 4′

The data were recorded at [a] 600 MHz and [b] 500 MHz; chemical shifts (δ) are in ppm, and coupling constants (*J*) are in Hz.

Table S3. NMR Data of Compounds **5** (in C_2D_6CO).

	**5** [d]		
**NO.**	δ_H (*J*)	**HMBC**	δ_c
1			82.9, CH
2	4.60, d (8.0)	C-3, 1', 2', 6', 4	68.6, CH
3	4.05, m		31.8, CH_2
4_α	3.00, dd (15.6, 5.2)	C-3, 2, 5, 9, 10	
4_β	2.66, dd (15.6, 8.8)	C-3, 2, 5, 9, 10	143.1, C
5			109.2, CH
6	6.42, d (2.4)	C-7, 8, a', 10	159.9, C
7			99.9, CH
8	6.27, d (2.4)	C-9, 7, 6	156.5, C
9			112.5, C
10			131.8, C
1'			111.9, CH
2'	7.04, s	C-2, 3', 6'	147.5, C
3'			143.1, C
4'			115.6, CH
5'	6.82, d (8.0)	C-1', 3'	121.5, CH
6'	6.89, d (8.0)	C-2, 2',3'	134.1, C
1''			113.1, CH
2''	6.83, s	C4'', 6'', a	148.2, C
3''			145.7, C
4''			115.7, CH
5''	6.74, d (8.0)	C-1'', 3''	121.7, CH
6''	6.69, d (8.0)	C-2'', a, 4''	37, CH_2
a	2.81, m	C-5, 1'', 6'', 10, a'	36.1, CH_2
a'	2.81, m	C-5, 1'', 6'', 10, a'	56.4, CH_3
3'-CH_3O	3.85, s	C-3'	56.3, CH_3
3''-CH_3O	3.81, s	C-3''	55.5, CH_3
7-CH_3O	3.72, s	C-7	
3-OH	4.00, brs		
4'-OH	7.60, brs		
4''	7.31, brs		

The data were recorded at [d] 400 MHz (^{1}H-NMR) and 100 MHz (^{13}C-NMR); chemical shifts (δ) are in ppm and coupling constants (*J*) are in Hz.
